# 3D-printed hip prostheses with regenerative integration: a state-of-the-art comprehensive review

**DOI:** 10.3389/fbioe.2025.1719810

**Published:** 2026-02-20

**Authors:** Sebastián Giraldo Gallego, Luis Eduardo Rodríguez Cheu

**Affiliations:** 1 BRIDGE-RISE Lab, GiBiome Research Group, Universidad Internacional de Valencia, Valencia, Spain; 2 GiBiome Research Group, Escuela Colombiana de Ingeniería Julio Garavito, Bogotá, Colombia

**Keywords:** 3D printing, biomaterials, hematopoietic stem cells, hip prosthesis, simulation

## Abstract

This article offers an in-depth review of the design and modeling of 3D-printed hip prostheses, emphasizing the integration of regenerative medicine, especially in the context of custom Rapidly Destructive Osteoarthritis implants. It traces the history of Total hip arthroplasty and current implant materials, along with recent advances in tissue engineering strategies and biofunctionalization methods to enhance biological integration. Computational processes are examined, including segmentation, image processing, computer-aided design, finite element analysis, and CAE simulations. It also discusses techniques in additive manufacturing that control porosity and stiffness, as well as strategies for recruiting host stem cells. The overall performance of existing THA approaches, combined with reliance on outdated surgical workflows and the complexity of clinical standards, creates challenges for the adoption of innovative implant research and limits broader application. International standards (ISO/ASTM), regional regulations (MDR, FDA), ethical considerations, and professional design guidelines are crucial components of this review, guiding safety, reproducibility, and the clinical impact of next-generation THA solutions. Finally, this review proposes a novel ‘regenerative design’ paradigm. Distinct from traditional biointegration methods, this framework integrates patient-specific imaging, mechanobiology-based architecture optimization, and biologically calibrated simulation to direct endogenous cell recruitment and vascularized healing explicitly.

## Introduction

Total hip arthroplasty (THA) has long been among the most successful orthopedic procedures, restoring mobility in patients with severe hip joint damage (Benignus et al., 2023; Creamer and Hochberg, 1997).

Over the decades, the procedure became routine, and although recent advances in technology and biology are opening new frontiers, progress in approaches to address the condition and bring about significant, high-impact changes in methodologies has been limited due to three main reasons: the medical environment and culture, regulatory issues, and clinical testing.

However, a critical gap persists in the management of complex hip pathology: the absence of a unified, mechanobiology-informed pipeline linking patient-specific imaging, architecture-driven design for endogenous cell recruitment, additive manufacturing, and validation. Motivated by these unmet clinical needs, this review synthesizes evidence for 3D-printed, patient-specific prostheses that support endogenous (autologous) stem-cell–mediated regenerative integration. Its goal is to justify this research, confirm its mechanistic plausibility, and present its potential as a groundbreaking direction for future research and clinical application by: (i) demonstrating its necessity, (ii) validating the scientific basis, and (iii) establishing a standardized methodology (routing). The review draws on prior studies and proposes new strategies to develop an efficient, rigorous approach to the design, simulation, and manufacturing of hip prostheses.

It begins with a historical overview of prosthetic development and then explores current materials and design approaches, including computational modeling and simulation tools. The section presents a case study of Rapidly Destructive Osteoarthritis (RDOA), along with image processing and selection, prosthetic strategies and innovations—comparing standard and custom designs, custom implant fabrication, emerging regenerative strategies, and our proposal that highlights the project’s potential and future outlook. Additionally, it addresses ethical, economic, and sustainability considerations. We also examine the role of medical imaging and segmentation in personalization, as well as the regulatory and ethical aspects of these innovations.

The goal is to demonstrate how an interdisciplinary approach (spanning engineering, medicine, and biology) can come together to develop the next-generation of hip implants by: (i) enhancing integration, (ii) addressing patient-specific clinical needs and conditions, (iii) proposing a standardized biomedical design methodology (guided pipeline), and (iv) establishing the foundational pillars for future research and innovation.

## Historical evolution of prosthetic limbs and hips

Human prosthetic technologies have evolved substantially over the centuries (National Library of Medicine, 2023). In the 20th century, external limb prosthetics advanced rapidly through improvements in control systems, materials science, and osseointegration, enabling more functional and responsive bionic devices (Bates et al., 2020).

By the early 21st century, emerging technologies such as virtual/augmented reality (VR/AR) and artificial intelligence (AI) were also being utilized in prosthetics–for example, to aid in design and enhance rehabilitation training (Bates et al., 2020; Wah, 2025).

Modern total hip replacements have undergone more gradual improvements than external limb prosthetics. The basic idea of THA–replacing the femoral head and acetabulum with a metal ball-and-socket joint–was transformed by Sir John Charnley in the late 1950s and 1960s (Hernigou, 2013; Gomez and Morcuende, 2005). Charnley’s low-friction arthroplasty used a small metal femoral head that moved on a polymer liner (originally ultra-high-molecular-weight polyethylene, UHMWPE) fixed with bone cement (Kumar et al., 2014), significantly enhancing the wear resistance and durability of hip implants and establishing the basis for modern THA.

Subsequent decades refined this proposal with improved biomaterials (e.g., cobalt-chrome alloys, titanium alloys, advanced polyethylenes), porous coatings for bone ingrowth, and designs optimized by clinical practice experience. Despite these advances, mainstream clinical practice has primarily relied on established solutions because of their proven safety. However, standard implants still face limitations in complex cases, where customized solutions must “prove their effectiveness and superiority” before widespread adoption (Benignus et al., 2023). As materials and precision have advanced, the fundamental biomechanics of THA have remained essentially unchanged. This historical pattern reveals a significant plateau: minor mechanical improvements have reached their limits, prompting a shift toward regenerative methods to address biological failures—such as aseptic loosening and bone loss—that traditional designs cannot fully address.

## Clinical challenge: rapidly destructive hip osteoarthritis

While conventional THA’s work well for common degenerative conditions, there are challenging cases that push current solutions to their limits because of the complexity, as they cannot be standardized due to variation from one patient to another.

One such condition is rapidly destructive osteoarthritis of the hip, or ‘RDOA’. RDOA^1^ is a rare^2^, atypical form of osteoarthritis (OA) (Chau et al., 2024) characterized by extremely rapid joint cartilage degeneration (studies begin indicating the condition when the annual joint space loss is ≥2 mm (Carrino et al., 2023) with no other apparent cause, which can progress to near-total destruction of the femoral head (St-Amant et al., 2024).

According to the National Institute of Arthritis and Musculoskeletal and Skin Diseases [NIAMS] (2023) reports, it is estimated that most patients with the condition are elderly women who can progress from a typical joint to near-complete destruction of the femoral head and acetabulum within 6–36 months (Batra et al., 2008).

Patient diagnosis of RDOA typically depends on clinical symptoms, including joint pain, stiffness, and a bone-on-bone friction sensation, combined with radiographic signs such as joint space narrowing (JSN) resulting from osteophyte formation (Hospital for Special Surgery [HSS], n.d.). This aggressive disease often results in significant bone defects in the hip (Çay et al., 2023).

Recent studies have highlighted the outcomes of THA in patients with RDOA. A systematic review by Baryeh et al. (2023) found that over 80% of RDOA cases required augmented reconstruction during hip replacement, including the use of bone grafts or cage implants to compensate for significant bone loss.

These surgeries were much more complex than standard THAs, with significantly greater blood loss (about 945 mL vs. approximately 580 mL in routine cases) and longer operative times.

The authors concluded that THA for RDOA involves “significant morbidity,” often requiring custom solutions and specialized fixation methods (e.g., cementless cups with augments, modular stems) to fill bone voids. They also highlighted a lack of long-term follow-up data on such cases and called for further research.

Another review (Chau et al., 2024) highlighted the importance of early detection of RDOA *via* imaging, as it “is a diagnosis not to miss” due to its rapid progression and the need for prompt treatment.

In short, RDOA describes a situation in which standard hip implants may not be sufficient and where advanced patient-specific and regenerative methods could be beneficial, as only custom prostheses can effectively restore function in these severely damaged joints.

This clinical motivation drives the exploration of innovative implant designs that promote bone regeneration and target specific conditions that may vary among patients (see Chau et al., 2024, for radiographic evidence of the extensive bone loss typical of RDOA patients).

This clinical urgency necessitates shifting from static replacements to dynamic, regenerative solutions. Consequently, the following sections explore the advanced materials and design strategies that enable this innovative approach.

## Materials used in hip prostheses

One key area of innovation in hip replacements involves the selection and development of biomaterials for implants.

The choice of material is crucial because it influences the implant’s mechanical strength, wear resistance, biocompatibility, and interaction with bone tissue. This directly affects the prosthesis’s overall performance and intended effect, as it can lead to complications and necessitate additional procedures.

For example, materials with significantly higher stiffness than bone can cause stress shielding, leading to bone resorption and increased prosthetic loosening (Mirza et al., 2010). Additionally, excessive wear produces debris that triggers inflammatory osteolysis and may require revision surgery (Morano et al., 2025; Wahbeh et al., 2022).

Now, the most used materials for THA interventions are presented in detail:

- Titanium Alloys (e.g., Ti-6Al-4V): Ti-based alloys are commonly used for femoral stems because of their excellent biocompatibility and resistance to corrosion. Among these alloys, Ti-6Al-4V is widely used due to its high strength-to-weight ratio; however, its elastic modulus (∼110 GPa) is approximately 5–10 times that of cortical bone (Ziaie et al., 2025). This stiffness mismatch can cause stress shielding, in which the stiffer implant bears excessive load, leading to resorption of the surrounding bone due to disuse (standards such as ISO 5832–3 specify the required composition and mechanical properties for implant-grade Ti-6Al-4V) (Morano et al., 2025).
o New manufacturing techniques, such as 3D printing, enable the production of titanium with porous lattices, effectively reducing its stiffness to improve compatibility with bone (Ziaie et al., 2025).
- Cobalt-Chrome Alloys: Co-Cr alloys are known for their strength and wear resistance. They are commonly used for femoral head or modular neck components because their hardness allows low-friction movement against a plastic liner or a ceramic. However, Co-Cr is quite stiff (with an elastic modulus of about 200 GPa) and relatively heavy, so it is less frequently used for stems.
o Co-Cr heads paired with modern liners offer excellent wear performance. However, concerns remain about the *in vivo* release of metal ions (Greenberg et al., 2021).
o Studies that measured Co and Cr levels in serum after CoCr–liner couplings (especially with CoCr–CoCr or CoCr–MoMo combinations) were conducted to analyze detectable concentrations of these compounds based on the head liners (Migliorini et al., 2023), along with assessments of modern prosthetics (dual mobility) and their conditions (Nam et al., 2017), as well as a review of local toxicity related to the systemic effects of wear debris (Bijukumar et al., 2018).
- Stainless Steel (316L): Austenitic stainless steel was commonly used in early orthopedic implants but is now less common.
o It offers good strength but is heavier and less resistant to corrosion compared to Ti or Co-Cr alloys. Stainless steel is mainly used in temporary implants like Kirschner wires, K-wires, and Steinmann pins for temporary fractures and external fixation, as well as skeletal traction (Uhthoff et al., 2006); or in historical designs such as Charnley’s early total hip prosthesis (Learmonth et al., 2007) and the original Austin Moore prosthesis (Willert et al., 2005), which have mostly been replaced by Co–Cr and titanium alloys.
- Ultra-High-Molecular-Weight Polyethylene (UHMWPE) and Highly Cross-Linked Polyethylene (HXLPE): UHMWPE has been the standard bearing material for acetabular liners since Charnley’s time, due to its low friction and biocompatibility (Charnley, 1961). However, conventional UHMWPE generates submicron wear particles in periprosthetic tissues (over years of use), where macrophages phagocytose them^3^ (Bozic and Ries, 2005), initiating an inflammatory cascade (Gupta, 2023)^4^ that ultimately stimulates osteoclast activity and leads to periprosthetic osteolysis (Harris, 1995).
o Highly cross-linked polyethylene (HXLPE) was introduced to significantly improve the wear resistance of acetabular liners dramatically (Kurtz et al., 1999).
    ⁃ Cross-linking involves exposing ultra-high molecular weight polyethylene (UHMWPE) to high doses of ionizing radiation, such as gamma rays or electron-beam, which causes covalent bonds to form between neighboring polymer chains. This bond network stabilizes the material, reducing the production of micro-wear particles during articulation with the femoral head (Muratoglu et al., 2001).
    ⁃ Following irradiation, thermal treatments such as annealing or remelting are used to quench free radicals and improve oxidative stability.
      ⁃ Annealed HXLPE maintains its crystalline structure and thus retains its tensile and fatigue properties; however, it may leave residual radicals that increase susceptibility to oxidation during storage and *in vivo* aging unless addressed (e.g., through sequential anneals or vitamin E stabilization).
      ⁃ Conversely, remelted HXLPE almost completely removes radicals and provides excellent oxidative stability (MacDonald et al., 2011). However, the melt-recrystallization process is linked to lower fracture and fatigue resistance and slight decreases in strength compared to annealed/Vit-E materials (Bracco and Oral, 2011; Kurtz, 2016).
    ⁃ These modifications convert conventional polyethylene into a more durable bearing material, significantly reducing wear rates *in vivo* and lowering the risk of particle-induced osteolysis (Dumbleton et al., 2002).
o Clinical data from the past 20 years show HXLPE liners have significantly lower wear and have effectively eliminated the osteolysis problem in modern THA (Morano et al., 2025).
- Ceramics (Alumina, Zirconia, and composites like ZTA): These materials are used either for the femoral head or the liner in some THA systems. They are extremely hard and scratch-resistant, providing the lowest friction among bearing couples.
o Third-generation ceramic-on-ceramic (CoC) bearings (Capello et al., 2008), often made from zirconia-toughened alumina (ZTA), have demonstrated very low wear rates and excellent long-term durability, especially in younger and more active patients (Hamilton et al., 2010). These advanced ceramics combine the hardness and scratch resistance of alumina with the transformation-toughening properties of zirconia, thereby enhancing fracture resistance relative to earlier ceramic types.
o Clinical studies and registry data consistently show that less osteolysis and implant loosening occur when CoC bearings are used in patients under 60 years old (Traina et al., 2011), making them one of the most reliable bearing options available today.
o The downside is the brittle nature of ceramics—earlier generations sometimes experienced sudden fractures—but modern formulations have reduced this risk.
o One of the most significant advantages of ceramics is the development of bioinertness, which is defined as the ability of a biomaterial to remain chemically and biologically inert within the body (Hench, 2006; Yu et al., 2015).
    ⁃ This means it neither causes harmful changes in the surrounding tissue nor undergoes significant alterations itself. In this way, the function of bioinert material depends on maintaining its structure and performance while passively coexisting with the host environment; therefore, it does not release metal ions.
  This set of conditions and features (especially ceramic heads made of HXLPE (American Joint Replacement Registry [AJRR], 2022)) made ceramic-bearing constructs a standard choice across many European centers.
    ⁃ Registry data shows that ceramic-on-polyethylene (CoP) is now the preferred option for THA in the UK, and ceramic femoral heads are standard in Germany (Endoprothesenregister Deutschland [EPRD], 2024). Adoption is also increasing outside Europe, with U.S. registry data showing that the most common primary THA designs involve pairing a ceramic head with an HXLPE liner (National Joint Registry [NJR], 2024).
  Polyetheretherketone (PEEK) and other advanced polymers: PEEK isa high-performance thermoplastic that, thanks to its remarkable Young’s modulus (∼3–4 GPa), is closer to cortical bone than metallic alloys, as well as its high resistance and low wear (Toth et al., 2006). It has proven to be worth testing in orthopedical implant design (Kurtz and Devine, 2007). The most interesting property of this advanced material is that it is radiolucent (does not appear on X-rays), which can aid in post-operative imaging (Kurtz and Devine, 2007).
    ⁃ One of the significant issues in THA follow-up is the presence of current metal implants (after surgical procedures), as they generate radiographic artifacts and obscure the bone-implant interface (Toth et al., 2006). This means that radiolucency facilitates more precise image visualization and improves the accuracy of rehabilitation assessments.
o PEEK is also lightweight and chemically inert. This characteristic of PEEK reduces its osseointegration potential and limits its regenerative effects in the joint following surgical intervention.
    ⁃ However, it has been demonstrated that its bioactivity can be improved by using techniques to modify surface (boundary) conditions and interactions with bone tissue through bioactive composites (Senra et al., 2023; Yu and Liu, 2021).
o Other advanced polymers, such as carbon-fiber-reinforced PEEK (CFR-PEEK), are being researched for additional orthopedic applications, including spinal instrumentation and THA-focused procedures (Brockett et al., 2017). Preclinical tribology studies have consistently shown better wear resistance under cross-shear conditions compared to unreinforced PEEK (Shi et al., 2022), and it is being evaluated for use in load-bearing implants such as hip cups, where CFR-PEEK hip implants have demonstrated the ability to achieve stable fixation *in vivo* (Nakahara et al., 2013).
  However, one long-term THA study (∼14.3 years of follow-up) reported mixed results for CFR-PEEK liners (Heijnens et al., 2020). Therefore, clinical data on PEEK in THA remain limited, and metal remains the gold standard for stems.

Additionally, Table 1 previews the key properties, limitations, and regulatory notes of the primary materials used in current THA operations and studies.

**TABLE 1:** This table summarizes the primary materials currently used in THA and their characteristics.

Material	Key properties	Limitations	Clinical/Regulatory notes	References
Titanium Alloys (e.g., Ti-6Al-4V)	Excellent biocompatibility, corrosion resistance, and high strength-to-weight ratio; widely used for femoral stems.	Elastic modulus approximately 110 GPa (5–10 times that of cortical bone) leads to stress shielding and a risk of bone resorption.	ISO 5832-3 specifies implant-grade Ti-6Al-4V; porous 3D printing can reduce stiffness.	Mirza et al. (2010), Morano et al. (2025), Ziaie et al. (2025)
Cobalt-Chrome Alloys (Co-Cr)	Highly durable with excellent wear resistance; low-friction articulation with PE/ceramics; commonly used for femoral heads.	Very stiff (∼200 GPa) and heavy; metal ion release (cobalt/chromium) can lead to adverse local tissue reactions.	Extensive clinical history: ASTM F75 outlines requirements.	Benignus et al. (2023), Morano et al. (2025)
Stainless Steel (316L)	Good mechanical strength and corrosion resistance.	Heavier and less corrosion-resistant than Ti/Co-Cr; less common in modern THA.	Historically widespread, it is now used mainly for temporary implants or in low-cost settings.	Mirza et al. (2010), Morano et al. (2025)
UHMWPE and HXLPE	Low friction and excellent biocompatibility; HXLPE = significantly reduced wear and osteolysis.	Conventional PE is susceptible to particle wear leading to osteolysis; HXLPE is more brittle than PE.	HXLPE is the standard of care for acetabular liners in modern THA	Morano et al. (2025), Ziaie et al. (2025)
Ceramics (Alumina, Zirconia, ZTA)	Extremely hard, scratch-resistant; lowest friction (ceramic-on-ceramic); bioinert	Early generations exhibited brittleness, which increased the risk of fracture. Additionally, some cases reported the occurrence of “squeaking.”	ZTA is a third-gen composite with toughness and it’s CE/FDA approved (preferred in young active patients)	Benignus et al. (2023), Morano et al. (2025)
PEEK and CFR-PEEK	The material is radiolucent, lightweight, and chemically inert. Its modulus ranges from 3 to 4 GPa, which is closer to that of bone. ; CFR-PEEK has higher strength	Limited long-term clinical data; mechanical limitations under high load conditions.	Under evaluation for cups and cages; ISO 10993 biocompatibility testing is mandatory.	Ziaie et al. (2025)
Porous Metals (Tantalum, Porous Ti)	A high surface area which facilitates osseointegration, while tunable stiffness and long-term biological fixation enhance implant performance.	More expensive; fatigue life needs to be optimized.	Used in revision cases, augments, cups; validated in RDOA defects	Baryeh et al. (2023), Ziaie et al. (2025)

Once candidate materials are reviewed, it is crucial to recognize that they must, before any *in vivo* or clinical use, meet strict mechanical and biological standards. These standards are established and verified within recognized regulatory frameworks (e.g., EU MDR, 2017/745, US FDA device regulations) and international standards issued by ISO, and ASTM, which collectively ensure safety, reproducibility, and functional reliability.

For total hip arthroplasty, this generally includes biocompatibility testing according to ISO 10993, hip wear simulation in accordance with ISO 14242, stem fatigue testing following ISO 7206, computational verification based on ASTM F2996, and system-level risk management testing per ISO 14971, all performed under a certified quality management system in line with ISO 13485.

The global regulatory environment, including competent authorities, notified bodies, and standardization organizations, addresses different aspects of device development and approval. These topics are covered in the following chapters: (i) Regulatory standards and mechanical testing requirements, and (ii) Ethical, economic, and societal considerations.

Key requirements for materials used in THA include having sufficient yield strength and fatigue endurance to withstand decades of cyclic loading, a modulus compatible with the host bone to minimize stress shielding, high wear resistance at the articulating surfaces, and biocompatibility to prevent toxic or inflammatory responses.

ISO 10993 standards are used to assess biocompatibility and ensure the absence of harmful local or systemic effects.

To establish the regulatory foundation for this framework, Table 2 consolidates the key international standards (ISO/ASTM) that govern material safety and mechanical performance. By summarizing the baseline requirements for fatigue testing, material composition, and biological evaluation, this table sets the mandatory ‘safety floor’ that any regenerative design must meet before addressing the pathology-specific needs of RDOA.

**TABLE 2:** Consolidated international regulatory standards (ISO/ASTM) governing safety and performance baselines for THA.

Framework/Standard	Purpose in THA	Key criteria/Tests	Min. acceptance
Eu MDR 2017/745	Regulatory framework for CE-marking in the EU	Clinical safety and performance; conformity assessment (NB review)	Clinical evaluation dossier supports intended use; benefit–risk balance is acceptable; PMS/PMCF plan is in place.
US FDA (21 CFR; QS/QSMR)	US device classification and quality system	Premarket pathway (510(k), *De Novo*, PMA); QMS requirements	Predicate equivalency (510(k)) or reasonable assurance of safety (PMA); compliant QMS
ISO 10993–1 (Biological Evaluation)	Biocompatibility of materials	Cytotoxicity, sensitization, irritation, systemic toxicity, genotoxicity	All necessary endpoints pass without adverse local or systemic effects; test matrix is justified based on risk.
ISO 14242 1/-2 (Hip wear)	Articulating surface wear simulation	Loading/displacement parameters; lubricant; gravimetric wear; particle analysis	The wear rate must be less than or equal to benchmark control, with no catastrophic damage; the particles are characterized.
ISO 7206–4/-6/-8 (Hip stem fatigue)	Fatigue endurance of femoral components	Quasi-static strength; fatigue testing to specified cycles/loads; endurance limit	Load–cycle curves, run-outs, failure modes; compliance with acceptance criteria
ASTM F2996 (FEA guidance)	Computational verification of implants	Model assumptions, mesh convergence, material models, boundary conditions, and validation	Verification and validation dossier (VandV), sensitivity analyses
ISO 14971 (Risk Management)	Systemic risk analysis/mitigation	Hazards, harms, probability/severity; risk controls and residual risk	Residual risks are acceptable and managed; complete traceability to design controls and verification.
ISO 13485 (QMS)	Quality management system for devices	Design control, CAPA, supplier, production, and process control	Certificate of conformity; includes audited QMS scope, covering THA production

**GRAPH 1:**
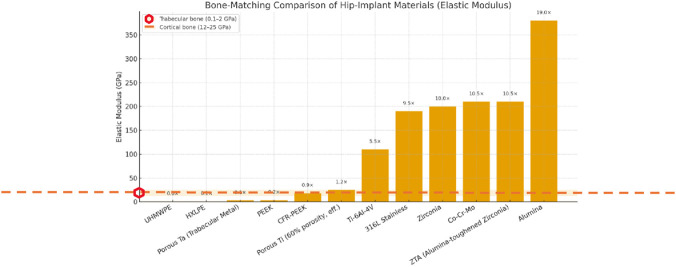
Comparative elastic modulus of hip implant materials *versus* human bone. The bar chart shows a noticeable stiffness mismatch between traditional solid metals (right) and the physiological ranges for trabecular bone (0.1–2 GPa) and cortical bone (12–25 GPa), as indicated by the shaded background areas. Note the better mechanical matching of porous tantalum, porous titanium, and advanced polymers (left) compared to rigid cobalt-chrome and ceramic options.

A comparison of elastic modulus implant materials from Table 1 (see Graph 1) between cortical (12–25 GPa)^5^ (Baleani et al., 2024; Ma et al., 2022) and trabecular (cancellous) bone^6^ (0.1–2 GPa) (Keaveny et al., 2001; Morgan et al., 2018) is displayed in Graph 1. The discrepancy shown explains stress shielding and subsequent bone resorption associated with hip implant materials, as clinically reported (Mirza et al., 2010; Morano et al., 2025).

The graph shows that porous titanium and CFR-PEEK fall within the expected effective modulus range for bone, resulting in fewer mechanical complications (stress dissipation), better surgical outcomes, and improved long-term osseointegration.

On the other hand, stiffness mismatch is a well-known and widely studied characteristic in the clinical context (Engh et al., 1987; Huiskes et al., 1992) of metals, as shown in Graph 1. This supports the evidence of stress shielding and subsequent bone resorption around the prosthesis.

**GRAPH 2:**
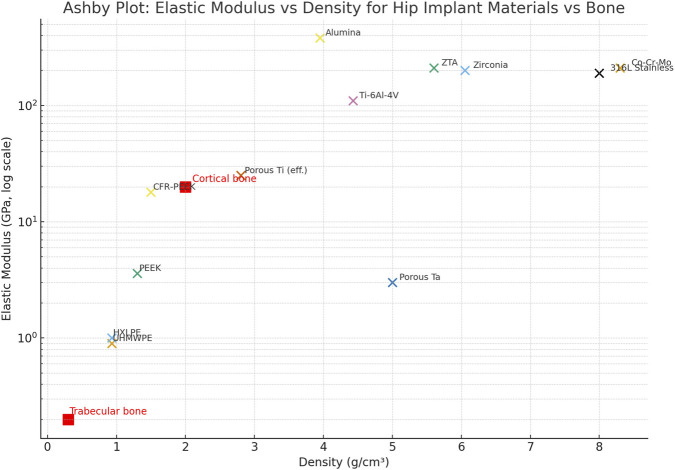
Ashby-style comparison of elastic modulus (log scale) *versus* density for hip-implant materials. The plot compares candidate materials from Table 1 with reference ranges for Cortical and Trabecular bone (shown as red squares). The distribution shows that advanced polymers and porous metal structures (grouped in the lower-left corner) move toward a more bone-matched property space. In contrast, conventional solid metals exhibit a significant mechanical gap.

An Ashby-style plot (Graph 2) displaying elastic modulus *versus* density illustrates the mechanical relationship between physiological bone and the candidate materials listed in Table 1. The data highlights the clear difference between the high stiffness and density of conventional metals (top right: Co-Cr-Mo, Stainless Steel) and the physiological target of cortical bone (∼20 GPa, ∼2 g/cm^3^). Notably, advanced polymers (PEEK, UHMWPE) and porous metallic structures (Porous Ti, Porous Ta) are located in the lower-left quadrant, clustering much closer to the physiological range.

This visualization emphasizes the potential of architecture-driven density reduction to reduce stiffness mismatch (Engh et al., 1987; Huiskes et al., 1992).

Based on the trends summarized in Graphs 1 and 2, recent research increasingly focuses on advanced polymers, porous metals, and bioactive coatings to overcome the limitations of traditional THA biomaterials. Notably, the data indicates that porous metal structures and advanced polymers more closely mimic natural bone mechanics than solid metallic designs. By reducing the stiffness mismatch that causes stress shielding, these material strategies create a mechanical environment better suited for durable load sharing and long-term biological fixation. The previous graphs indicate that recent research emphasizes advanced polymers, porous metals, and bioactive coatings, given the recognized needs and limitations of current biomaterials for THA. For instance, porous tantalum and 3D-printed lattice structures in titanium enhance osseointegration while reducing the overall stiffness of the implant (Bobyn et al., 1999; Levine et al., 2006; Murr et al., 2010). Another example is the use of porous titanium coatings on stems and acetabular shells to achieve long-term biological fixation (Ziaie et al., 2025).

Therefore, engineered porous architectures can be designed to facilitate cell seeding, bone ingrowth, and improved osseointegration while adjusting the mechanical stiffness to match biological requirements (Hollister, 2005; Karageorgiou and Kaplan, 2005; Ryan et al., 2006).

As discussed in this section, materials illustrate the evolution of implant research over decades: from the early reliance on standard 316L stainless steel in response to THA to the integration of materials science into regenerative principles to address specific conditions and pathologies, such as RDOA.

In conclusion, each THA implant material in the medical portfolio used for design purposes has its own trade-offs.

Each material, along with its associated advantages and limitations, is summarized: the clinical standard for enhancing osseointegration is achieved with porous Ti/Ta or bioactive coatings. Ceramics exhibit very low wear resistance, despite being brittle. The maintenance process for UHMWPE/HXLPE low-friction bearings requires precise monitoring of their wear levels. The PEEK/CFR-PEEK material shows radiolucency and a bone-like modulus, but surface modification is necessary to ensure proper bonding (with variable long-term hip results). Optimal implants feature material properties that enable the fabrication of biomaterials with specific properties through a design approach tailored to individual musculoskeletal shapes and body measurements (Hollister, 2005; Murr et al., 2010; Ryan et al., 2006).

All these elements lead to the standardized and widely recognized THA implant design, summarized as follows: a metal stem for strength, a ceramic or metal head for low wear, and a polyethylene liner for cushioning, a pattern that is reflected across contemporary national registries (American Joint Replacement Registry [AJRR], 2022; Endoprothesenregister Deutschland [EPRD], 2024).

## Medical imaging and segmentation for implant design

The initial step in designing a personalized hip prosthesis is to obtain medical images that capture the patient’s unique body shape and anatomy.

The standard assessment process for THA includes computed tomography (CT) scans, which are used to evaluate bone structure and to generate models for component templating and robotic or navigation systems. Magnetic resonance imaging (MRI) offers additional information about cartilage, labrum, marrow, and soft tissues when radiographs are insufficient (Jawetz et al., 2023). The volumetric data then undergoes processing, including segmentation, surface reconstruction, and mesh cleanup, to produce 3D digital models that surgeons and engineers use for planning and implant development (Fontalis et al., 2024; Lu et al., 2025; Mozafari et al., 2024).

The existing hardware requires metal artifact reduction (MAR) methods, such as dual-energy, algorithmic, and deep learning-based MAR, to enhance visualization (Selles et al., 2024).

Regarding international standards for image processing and reproducibility, all imaging data and models must comply with the Digital Imaging and Communications in Medicine (DICOM) standard, which is the global standard for secure, anonymized information exchange and the protection of patients’ sensitive data (National Electrical Manufacturers Association [NEMA], 2023).

Image segmentation is the core step of medical imaging analysis, involving the delineation of relevant anatomical regions (e.g., bone, cartilage) in CT or MRI data.

The traditional approach to segmentation used manual tracing or basic thresholding, which were slow and operator-dependent, leading to inconsistent results in pathological identification and interpretation. Advances in technology and the growing acceptance of AI have significantly improved these methods, now supporting this process.

The U-Net architecture, along with other deep learning models, achieves state-of-the-art performance in biomedical segmentation tasks by learning to identify anatomical structures at the pixel or voxel level. Ronneberger et al. (2015) developed the U-Net architecture for biomedical image segmentation, which led to various U-Net variants, including the 3D U-Net, that yield outstanding results for lymph node detection in CT images (Çiçek et al., 2016; Zhou et al., 2019).

In hip prosthesis design, U-Net–based architectures can be trained to automatically segment the femur and pelvis from CT scans, including pathological areas such as bone loss or cystic defects, resulting in highly accurate 3D reconstructions of the patient’s anatomy. This automation not only shortens processing time but also improves reproducibility compared to manual workflows.

Indeed, AI algorithms have already achieved radiologist-level accuracy in many segmentation tasks and continue to improve as larger, curated training datasets become available (Çiçek et al., 2016; Isensee et al., 2021; Ronneberger et al., 2015).

In clinical and research settings, however, segmentation is still often performed using dedicated open-source platforms, such as ITK-Snap and 3D Slicer (Fedorov et al., 2012; Yushkevich et al., 2006). Training data for these tasks are increasingly sourced from large repositories such as TotalSegmentator and CTPelvic1K, which offer annotated CT datasets for reproducible orthopedic research (Isensee et al., 2021).

Given the focus of this review, which aims to propose a guideline directly addressing RDOA, it is appropriate to establish criteria and specific requirements for image segmentation that align with this focus.

The characteristics of patients with RDOA, as examined in the clinical challenge, baseline radiographs, and scheduled comparisons to monitor progress, are necessary (Rosenberg et al., 1992). Additionally, CT is used to detect possible subchondral fractures and to map bone loss. On the other hand, MRI is crucial for early detection (Boutry et al., 2002) of signals similar to edemas and effusion/synovitis (excluding mimics^7^) (Chau et al., 2024; Shu et al., 2014).

Once the geometric condition is established through image analysis, it is important to quantify the defects to properly design the next implant (Paprosky et al., 1994). Classifying acetabular bone loss is crucial for guiding the use of augments and addressing pelvic discontinuity (image records based on categorized THA patients vs. segmental case defects) (Telleria and Gee, 2013).

Additionally, selecting the fixation strategy and stem geometry is crucial; therefore, cortical support mapping and femoral canal taping are recommended.

Next, patient-specific planning, focusing on alignment and stability, is established (Lewinnek et al., 1978) by predefining cup orientation and performing range-of-motion checks. While it is recommended to use safe zones (expected or desired inclination and anteversion angles) as a starting reference, spinopelvic parameters should be tailored to the individual (Tezuka et al., 2019). The necessary implant must be designed based on fixation biology identified *via* image processing, ensuring sufficient bone stock is available, and aiming for micromotion of less than 150 μm to promote bone ingrowth (Kohli et al., 2021).

Ultimately, imaging data should be the primary focus, as comprehensive parameterization in DICOM, along with proper settings in document segmentation and quality metrics for regulatory compliance, guarantees traceability across different centers (Anitua et al., 2015).

Building on Figure 2 in Manjunatha et al. (2023), we present a Routing-First Imaging and Segmentation (RFIS) workflow as a standardized quality-gate system for hip CT studies of complex hip conditions.

Instead of viewing segmentation as merely a technical step, RFIS redefines imaging and segmentation as a controlled, defect-aware starting point in the regenerative design process. Its key innovation is combining (i) anatomical masking (e.g., cysts, bone loss, and subchondral plates); (ii) clear multi-stage quality checks to verify imaging suitability and segmentation accuracy; and (iii) automated geometric quantification that converts validated masks into discrepancy maps and watertight 3D meshes for downstream CAD/FEA.

This approach aims to ensure consistent inputs across sites, minimize operator-dependent variability, and produce robust anatomical models suitable for RDOA-specific parametrization while reducing early-stage error propagation into design and simulation.

### Method (RFIS SOP—Condensed)

#### Stage 0—image routing and selection (pre-AI)

o Inclusion criteria: Hip/pelvic CT studies must meet predefined acquisition standards, such as slice thickness ≤1.0–1.25 mm, near-isotropic voxel geometry when available, complete anatomical coverage of the hemipelvis and proximal femur, and DICOM datasets with preserved metadata. Studies with severe motion artifacts or uncontrolled metal artifacts are excluded or rerouted for metal artifact reduction (MAR) protocols when clinically necessary and feasible. The Quality control (QC): a five-domain checklist is applied prior to segmentation: (i) signal-to-noise ratio (SNR); (ii) HU consistency/calibration stability; (iii) anatomical coverage; (iv) artifact severity score; and (v) de-identification verification.

Only datasets that meet all thresholds proceed to Stage 1. Nonconforming datasets are returned to inventory for multisourcing replacement or re-acquisition. Multisourcing and pipeline validation requirements are outlined in the Image Routing document, while project-specific CT acquisition parameters are detailed in each study proposal.

#### Stage 1—defect-aware segmentation and classification

This stage performs automated defect-aware segmentation using a two-stage U-Net/CNN architecture. A 3D/2.5D U-Net creates candidate masks for the pelvis and femur, along with lesion proposals that identify bone loss, cysts, and subchondral plate involvement. A lightweight 3D CNN classifier then validates lesion candidates and filters out isolated or low-confidence findings to enhance specificity in complex joint pathologies.

The training approach uses class-balanced patch sampling and standard augmentation techniques (rotation, scaling, elastic deformation), combined with lesion-aware loss functions (Dice and BCE).

Quality assurance is performed through expert review of specialized 3D medical imaging software, such as 3D Slicer or ITK-SNAP, using standardized naming conventions and version-controlled exports. Internal performance standards require Dice scores ≥0.90 on test cases and adherence to pre-defined KPIs.

o Relevant algorithmic advances in pathology-sensitive imaging optimization (e.g., recent approaches applied to complex MRI segmentation) are identified as complementary directions for future refinement of multimodal RFIS extensions (Song and Yang, 2023).

#### Stage 2—mesh and anatomical metrics

o Mesh generation: validated segmentation masks are transformed into high-fidelity 3D anatomical models using the marching cubes algorithm.

Post-processing steps include watertight mesh generation, correction of non-manifold edges, and tolerance-controlled smoothing, with final export as STL/PLY files suitable for CAD/FEA.

o Defect quantification: to enable defect-aware planning, the system creates standardized discrepancy maps and metrics.

Key outputs quantify cortical thinning (% area change), bone loss volume (mL), maximum defect depth (mm), and cyst count/size distribution.

o Design integration: when baseline imaging is available, the workflow calculates Δ-joint space measurements.
o Together, these outputs convert imaging pathology into consistent numerical design constraints, acting as direct inputs for RDOA-specific design goals and validation thresholds.

#### Stage 3—outputs and acceptance gates:

o Validation metrics: segmentation accuracy is confirmed using strict quantitative thresholds, including global and lesion-specific Dice scores, 95th-percentile Hausdorff Distance (HD95), and per-label precision and recall.
o Mesh integrity: final surfaces undergo automated quality assurance to verify watertightness, meet minimum triangle quality thresholds, and ensure consistent surface normals.
o Clinical output package: the final release consolidates surgeon-approved discrepancy readouts, the completed Image Routing form, and evidence of QC clearance.

These documents should be attached to the patient case file to create a traceable, audit-ready record that supports downstream CAD/FEA and translational process validation.

To ensure full auditability, RFIS maintains strict traceability by storing raw DICOM inputs, routing and QC logs, version-controlled model versions, and export-integrity checks (including cryptographic hashes where applicable). This organized data lineage allows verification of every subsequent design decision—from initial defect-aware segmentation to final mesh approval—against established quality gates.

**FIGURE 1:**
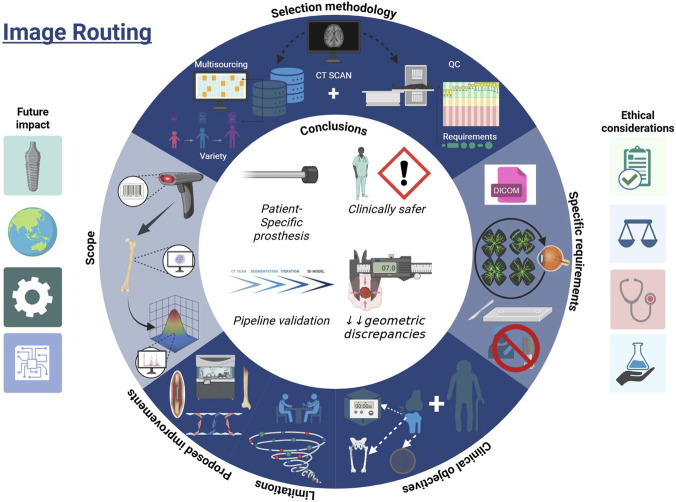
Integrated image routing and quality framework (RFIS). The schematic outlines the centralized workflow for transforming raw imaging data into patient-specific design inputs. The cycle enforces standardized Quality Gates (top/right quadrants) covering multi-source CT selection, strict QC auditing (SNR/Artifact scoring), and DICOM metadata preservation. This routing logic links upstream clinical goals with downstream validation metrics, ensuring that every segmentation pipeline is reproducible, auditable, and clinically safer by reducing geometric discrepancies before manufacturing.

An illustrated overview of this integrated framework is shown in Figure 1. While the text explains the specific criteria for each stage, this diagram visually represents the standardized decision logic, showing how clinical objectives and ethical considerations (right) influence the selection and routing process (top) to ensure reproducible, audit-ready inputs for the design pipeline.

## Computational modeling and finite element simulation (REGENERATIVE DESIGN)

The patient-specific anatomy model enables virtual prototyping using computer-aided design (CAD) and finite element analysis (FEA).

The segmented pelvis and femur generate a geometric discrepancy map that highlights local area changes, cortical thinning, cavitary or segmental bone loss, and complex curvatures, aiding in the assessment of implant fit, surface, and load pathway.

We also observe a growing trend among researchers toward regenerative design: an innovative approach that combines patient-specific CAD, FEA, and AM techniques to create personalized porous structures that restore physiological bone loading and enhance tissue bonding.

The review establishes acceptance criteria through practical implementation, including mesh convergence, zone-wise micromotion, fatigue margins, wear targets, remodeling outputs, and porous-transport metrics, before transferring detailed formalization to future research.

First, the implant geometry is modeled in CAD software (e.g., Autodesk Inventor, SolidWorks, or FreeCAD). The design is then morphometrically registered (based on the image segmentation discussed previously) to establish press-fit regions and define matching interfaces, such as those intended to accommodate porosity. Using the segmented bone as a reference, the implant is shaped for a close, patient-specific fit.

For a custom RDOA stem, it is suggested to (i) use canal-conforming stems with controlled cross-section transitions to prevent stress risers, (ii) include flanges or buttresses that bridge segmental defects, and (iii) incorporate porous contact zones aligned with the remaining high-quality bone stock.

Modern CAD pipelines support algorithmic and generative design to optimize topology. Simultaneously, periodic or graded lattice architectures (unit cell size, shape, porosity) are parameterized to achieve a target effective modulus and maximize osseointegrative surface (Gibson and Ashby, 1997; Hollister, 2005; Murr et al., 2012; Ryan et al., 2006).

Therefore, the volume data are converted into a patient-specific 3D surface mesh—most often using the marching cubes algorithm to extract an isosurface from CT/MRI voxels (Lorensen and Cline, 1987)—and then, if necessary, by Poisson surface reconstruction to create a watertight model suitable for downstream CAD/FEA applications.

The mesh is then cleaned and quality-checked, including hole filling, removal of non-manifold elements, curvature-aware smoothing, and controlled decimation, aiming for element quality suitable for analysis and manufacturing (e.g., limits on aspect ratio/dihedral extremes and voxel-matched edge lengths in surgical regions) (Kazhdan and Hoppe, 2013).

Iterations are essential in FEA studies because they allow the simulation of various physiological boundary conditions with minimal computational effort, making them indispensable in preclinical research and a valuable tool. Common boundary conditions, as outlined in regulations such as ASTM F2996, ISO 7206–4/-6/-8, and ISO 14242, include evaluating micromotion at bone–implant interfaces, peak stresses in weakened cortices around defects, and fatigue safety factors for worst-case activities (ASTM International, 2024; International Organization for Standardization [ISO], 2010; International Organization for Standardization (ISO), 2014; International Organization for Standardization (ISO), 2015; Kohli et al., 2021).

The model’s optimization involves adjusting lattice parameters and graded densities within functional biological ranges, specifically cell size (300–800 μm) and porosity (50%–75%). These adjustments may then be fine-tuned to minimize stiffness mismatch while maintaining primary stability (Hollister, 2005; Ryan et al., 2006).

Once a design is drafted, finite element analysis is employed to simulate its mechanical performance.

A typical simulation for a hip stem might replicate forces from walking, stair climbing, or stumbling, while ensuring that stress hotspots in the implant remain below the material’s fatigue limit. It also assesses the load transfer to the bone. Gradually varying sectional areas enable polar behavior in the stem, with a stiff proximal section for initial stability and a more flexible distal section to facilitate bone load sharing.

FEA can be used to compare a solid stem with a lattice stem, demonstrating how the lattice reduces stress on the adjacent bone by deforming more under load (Ziaie et al., 2025). It can also forecast micromotion at the bone-implant interface, helping assess whether initial fixation will be stable, as excessive micromotion can hinder bone ingrowth.

In the design of a regenerative implant, simulations can also evaluate fluid flow and the porous network’s permeability, as these factors may affect stem cell migration and nutrient transport within the implant. Further research into this behavior could help expand the presented hypothesis.

When finite-element analysis is required, the surface is tetrahedralized with size fields that refine near interfaces, porous zones, or screw paths, and Jacobians and skewness are verified to prevent inverted or poorly conditioned elements (Yu et al., 2014).

These steps create an accurate digital twin of the patient’s hip, allowing planning software to simulate implant size, position, and orientation.

Simulations for RDOA should grade cross-sectional transitions with adequate length to help reduce stress concentrations. Specifically, the lattice–solid junctions are equipped with fillet radii to minimize local peak stresses.

Critically, this computational verification is essential for predicting and preventing high-cycle fatigue failure of the porous struts, a standard failure mode in load-bearing lattice structures (Zadpoor, 2019).

Beyond mechanical stability, computational analysis extends to the biological interface. Specifically, a Darcy-type flow/permeability analysis of the regenerative design’s porous network examines the hydrodynamic transport of cells and nutrients through the lattice.

Model credibility is shown through mesh-convergence testing (h-refinement until stress/micromotion changes fall below a set tolerance), contact-parameter sensitivity (friction, tie *versus* sliding), and focused parameter sweeps on lattice density and transition geometry (following best-practice FEA guidance).

Assessing static structures is just one aspect of simulation activities. The simulation model should incorporate stress and micromotion analyses, articulation wear modeling using multi-body dynamics or FEA-based wear laws, and mechanobiological bone remodeling to predict density changes caused by altered load paths (ASTM International, 2024; Huiskes et al., 1992; International Organization for Standardization (ISO), 2014).

Complex models enable predictions about bone adaptation patterns, supporting the regenerative goal of developing an integrated implant system that encourages bone growth in targeted areas while preventing bone loss.

The models produce standardized results using predefined acceptance criteria that include (i) stress and interface micromotion convergence with mesh refinement showing less than 5% change (ASTM International, 2024), (ii) primary stability with micromotion levels below 50–150 µm on designated ingrowth surfaces (Kohli et al., 2021), (iii) fatigue safety with peak alternating stress values below the allowable threshold and a minimum 20% safety margin in worst-case loading conditions (International Organization for Standardization (ISO), 2010; International Organization for Standardization (ISO), 2015), (iv) wear performance that achieves target volumetric wear rates during standardized testing cycles (International Organization for Standardization (ISO), 2014), (v) remodeling predictions that prevent significant bone-mass loss in risk areas below 10%–20% at 12–24 months equivalent time points (Huiskes et al., 1992), and (vi) transport suitability evaluation using permeability (k) and superficial velocity measurements for nutrient delivery in porous regions (Hollister, 2005; Ryan et al., 2006).

Crucially, in this regenerative design context, simulation outputs are viewed as a connected set of biologically calibrated performance variables rather than just mechanical safety indicators. Specifically, interface micromotion is evaluated as a region-specific stability window that encourages early cell adhesion and osseointegration while lowering the risk of fibrous encapsulation. Stress distribution and load transfer are optimized to restore natural strain patterns and reduce stress shielding, supporting bone maintenance and remodeling in targeted areas. Additionally, permeability and transport metrics are used to assess whether the porous network permits marrow-accessible nutrient flow and cellular migration, both of which are important for angiogenesis and the proposed enhancement of HSC-supported niche reconstitution.

Accordingly, architecture-driven adjustments—such as lattice grading, unit-cell morphology, porous-zone thickness, and transition geometry—are iteratively refined to match these three outputs with both mechanical strength and regenerative goals.

Utilizing stiffness-matched architectures in medical implants may help reduce stress shielding while maintaining primary stability, according to existing research on lattice structures and mechanobiology (Murr et al., 2010; Vafaeefar et al., 2025).

A comprehensive regenerative design framework that accounts for lattice fatigue at junctions, transport suitability of porous regions, and standardized remodeling targets is beyond the scope of this paper. We present the key criteria we used for credibility assessment and recommend further research to develop standardized metrics, validation methods, and datasets.

The development of this work (if proven to be effective and methodologically sound) could establish standardized benchmarks for regenerative implants, thereby promoting consensus within the field.

## Additive manufacturing (AM) in custom patient-specific implant design

The standard procedure for THA involves surgeons selecting implants within predefined sizes, using standard designs, and performing bone reaming and cement application to ensure a proper fit for each patient.

Custom designs provide patients with better fit and improved biomechanical restoration, enabling handling of complex anatomical cases and significant defects that standard implants cannot address (Benignus et al., 2023). Standard hip implants suit most patients, but custom implants are mainly used in complex cases involving severe dysplasia or tumor reconstruction when standard implants cannot achieve proper contact or alignment.

3D printing technology enables the fabrication of customized implants that precisely match the shape of both acetabular defects and deformed femurs. Research indicates that these patient-specific devices enhance implant placement accuracy and joint function; however, further studies are needed to fully understand their impact on patients (Benignus et al., 2023).

Developing a personalized implant presents two main challenges: it is time- and cost-intensive, often limiting its availability to rare cases. However, advancements in sophisticated CAD software and AM (industrial 3D printing) have made the process more accessible. Today, custom hip implants can be produced in days using techniques such as electron-beam melting or selective laser melting of metal powders.

This review aligns with recent literature and fully follows a patient-specific AM workflow: imaging → segmentation → patient-specific 3D model → CAD/FEA optimization → AM manufacturing.

Producing complex, patient-specific features with graded lattices and anatomical contours is not feasible with traditional subtractive machining or forging; however, AM enables the direct fabrication of such components.

The Food and Drug Administration [FDA] (2017) states that lattice-inlaid stems with periodic or graded architectures can be printed as monolithic structures, and porous bone-facing surfaces can be integrated during the build process, thereby eliminating the need for secondary coating steps.

Modern powder-bed fusion (PBF) systems achieve layer resolutions on the order of tens of micrometers (typical laser powder bed fusion [LPBF] ∼20–60 µm and electron beam melting [EBM] ∼50–100 µm) (Taghian et al., 2023; Murr et al., 2012), which is sufficient to produce bone-ingrowth pore sizes in the range of ∼300–800 μm, as recommended for osteogenesis and vascularization when combined with suitable unit-cell design and post-processing (Karlsson, 2015).

Achieving high geometric fidelity remains essential; advanced modeling techniques, such as height difference-based profiling, are increasingly used to improve droplet deposition and surface quality in drop-on-demand printing systems (Wu and Chiu, 2021).

Using a fully custom design increases the contact area between the implant and bone, resulting in immediate stability and improved load transfer. The procedure is less complex because patient-specific implants require less bone removal than standard implants, as they match the exact dimensions of the existing bone structure. The surgeon uses custom guides and jigs (including 3D-printed versions) to prepare bones for implant placement during complex surgical procedures.

Patient-specific implants yield better outcomes in specific clinical settings due to their unique combination of factors. In fact, initial cases have demonstrated that custom implants can provide stable fixation and good function, even in patients with unique anatomies that would otherwise be challenging (Baryeh et al., 2023).

Standard medical procedures rarely use custom implants as a treatment option. They tend to be more expensive, and regulatory approval is typically granted on a case-by-case basis, such as through a custom device exemption in some regions.

Developing a custom implant becomes more difficult when a patient requires revision surgery, as there is no universal implant to revert to. The surgeon must design a custom device for the revision because a standard implant is unavailable.

The factors mentioned have limited the use of patient-specific hip prostheses to research settings and rare cases in RDOA studies. However, as manufacturing costs decline and the digital design-to-production process improves, personalization is likely to become more common.

Starting each design from a blank slate enables designers to create unique shapes and incorporate new features, such as personalized porosity and bioactive reservoirs. The final product is a customized hip implant tailored to each patient, aligning with the current trend in precision medicine in healthcare.

As shown throughout the review, both early evidence and biomechanical theory indicate that, when properly manufactured, custom biomanufactured AM implants can provide more natural load distribution and potentially greater durability by avoiding the compromises associated with off-the-shelf devices.

To implement the proposed regenerative design framework, Table 3 clearly compares established design parameters used in standard custom THA with RDOA-specific constraints proposed here. While conventional protocols focus on generic anatomical fit and overall fixation targets, the right column of Table 3 emphasizes key contributions of this review—most notably the shift from geometric segmentation to lesion-aware masking (e.g., classifying cyst burden and subchondral plate integrity) and the use of zone-specific micromotion thresholds.

**TABLE 3:** THA 3D design (generalized) vs. RDOA-focused parametrization.

Established pipeline	THA 3D design – generalized	RDOA-focused parametrization (specific)
Primary imaging	CT (≤1–1.25 mm) for bony morphology; MRI optional for soft tissue (Rosenberg et al., 1992; Boutry et al., 2002; Chau et al., 2024)	Baseline + serial radiographs for rapid change; CT for subchondral fracture and bone-loss mapping; MRI for edema-like signal/effusion and to exclude mimics
Segmentation labels	Pelvis, femur (Yushkevich et al., 2006; Fedorov et al., 2012; Isensee et al., 2021; Deng et al., 2022)	Pelvis, femur + bone-loss/cysts, subchondral plate, head collapse markers
Repositories/tooling	3D Slicer/ITK-Snap; optional AI (U-Net/nnU-Net)	Same, plus lesion-aware training or class-balanced patches to capture defects
Geometric descriptors	Canal centerline; cortical shell; head center; anatomical frames	Discrepancy map (area change %, cortical thinning, cavitary/segmental loss), pelvic continuity flag
Quantification outputs	Offsets (FO/GO), leg length, version, cup inclination/anteversion	Bone-loss volume (mL), max defect depth (mm), cyst count/size, joint-space Δ vs. prior
Defect classification	Standard acetabular/femoral assessments (Paprosky et al., 1994; Telleria and Gee, 2013)	Paprosky acetabular class, segmental vs. contained defects, pelvic discontinuity decision
CAD objective	Fit and alignment for standard components.	Anatomic reconciliation around defects; define press-fit islands on remaining strong bone
Fit strategy	Canal-conforming stem; standard shell	Flanges/buttresses/augments bridging segmental loss; canal fit with controlled section transitions
Surface/porous strategy	Optional porous coating for biological fixation	Targeted porous contact zones on viable bone; graded lattice for stiffness tuning
Porous parameters	Pore 300–800 μm; 50%–75% porosity (typical) (Hollister, 2005; Ryan et al., 2006)	Same ranges, regionally tuned to hit bone-like effective modulus near defects
Topology/generative design	Weight reduction while maintaining strength	Load-path restoration around defects; protect thin cortices (stress-riser avoidance)
FEA boundary conditions	Physiologic joint and muscle loads; gait cycle envelopes (Lewinnek et al., 1978; Tezuka et al., 2019)	Same, plus defect-edge stress audit, screw-trajectory pull-out, pelvic-ring continuity checks
Primary stability target	Interface micromotion <50–150 µm for ingrowth (Kohli et al., 2021)	Same threshold, reported per contact zone adjacent to defects
Modulus alignment	Reduce stiffness mismatch where feasible (Gibson and Ashby 1997; Murr et al. (2012)	Explicit lattice grading is required to maintain adequate local elasticity within the specified trabecular and cortical stiffness windows.
ROM/impingement	Global ROM with bony/implant impingement; Lewinne safe zone as start	Same, individualized by spinopelvic parameters; edge-loading risk heightened by defects
Fixation and screws	Standard safe corridors (ASTM International, 2024; International Organization for Standardization (ISO), 2010; International Organization for Standardization (ISO), 2014)	Pre-planned screw corridors to bridge discontinuity/augments; minimum cortical purchase defined
Manufacturing constraints	Strut ≥300–500 μm; support strategy; HIP/post-process	Same, plus tolerance plan across hybrid regions (solid–lattice–coating) and defect-facing fits
Validation and outputs	STL/STEP models; bench fit on printed bone	Lesion-aware metrics (Dice/HD95 for defect masks), micromotion maps, and surgeon review of defect reconciliation
Documentation/QA	DICOM preserved; segmentation/mesh SOPs; QMS traceability (National Electrical Manufacturers Association [NEMA], 2023)	Same, with defect logs (IDs, volumes, depths), MAR settings, and zone-wise acceptance gates

The table provides a simplified overview of the complete THA implant design process, including imaging, segmentation, modeling, CAD/FEA, AM, verification, and documentation, and compares it to the specific design requirements for RDOA cases. The right column highlights the new requirements and enhanced quality-control measures (defect-aware imaging, lesion labels, discrepancy mapping, lattice grading, zone-wise micromotion), based on the primary standards used as evidence.

Collectively, these parameters signal a shift from purely geometric fitting toward pathology-responsive design. By adjusting local mechanics to safeguard compromised bone stock, this framework aims to transform the implant from a passive mechanical spacer into an active driver of biological processes repair.

## Integration of regenerative medicine in implant design

Perhaps the most innovative feature of the 3D hip prosthesis being reviewed is its regenerative integration. The design aims not only to replace damaged bones but also to stimulate the body’s active healing and tissue regeneration processes.

Traditional implants are passive; they provide support but rely on the body’s natural healing processes—such as bone growing onto a porous surface—for fixation. In contrast, this new approach intentionally utilizes principles of regenerative medicine by creating an implant that interacts with the patient’s biology in a more instructive way.

The key feature is a porous structure specifically designed to attract the patient’s own stem cells. The prosthesis is 3D-printed with a network of interconnected pores and channels that enable the migration of bone marrow and cells into the implant. In fact, an implant that can enable the migration and colonization of hematopoietic stem cells, thereby facilitating tissue integration and regeneration.

In other words, beyond just promoting bone ingrowth, the goal is for blood-borne progenitor cells—particularly hematopoietic and mesenchymal stem cells from the marrow—to populate the implant’s porous scaffold.

Once there, these cells can differentiate into bone-forming osteoblasts or other supportive cells, contributing to the formation of new bone tissue within and around the implant. Essentially, the implant serves as both a mechanical device and a tissue-engineering scaffold.

### Why HSCs over MSCs?

The rationale for targeting Hematopoietic Stem Cells (HSCs) lies in their unique ability to link osteogenesis with angiogenesis, releasing factors that coordinate blood vessel growth and immune regulation (Grosso et al., 2017). It is important to recognize that Mesenchymal Stem Cells (MSCs) exhibit greater intrinsic osteogenic capacity and are the primary drivers of direct mineralized matrix formation (Peng et al., 2020). However, MSC-driven repair often yields inconsistent outcomes in ischemic environments with reduced vascular support.

Therefore, this review presents an HSC-centric approach not as a substitute for MSC osteogenesis but as a proposed “vascularization-first” mechanism. We suggest that, in the specific context of RDOA—characterized by rapid bone loss and vascular damage—HSCs may serve as essential upstream architects, restoring the vascular niche required for subsequent MSC engraftment and ongoing remodeling (Ramasamy et al., 2016).

This distinction positions the HSC strategy as a specific hypothesis for vascularized niche reconstitution rather than as a proven alternative to direct bone formation.

To substantiate any claims of superiority, future studies must directly compare HSC, MSC, and HSC + MSC co-culture models using lineage tracing, quantitative analysis of vascular density, and mechanical outcome measures (Jia et al., 2021; Vinci et al., 2023). Previous work has established a foundation for this concept. Porous coatings, such as hydroxyapatite, and bioactive surface treatments have been used for decades to enhance osseointegration (LeGeros, 2002; Karia et al., 2023).

Experimental scaffolds seeded with stem cells for orthopedic repair also exist; however, these are typically temporary bone graft substitutes rather than permanent load-bearing implants (Vidal et al., 2020). To our knowledge, based on the comprehensive literature analysis conducted for this review, no prior hip prosthesis design explicitly aims to use endogenous stem cells as part of its functional mechanism.

A comprehensive review by Ziaie et al. (2025) on advanced porous hip implants highlights that pore size and structure can be tailored to enhance tissue ingrowth and mechanical properties.

For example, pores approximately 500 microns in diameter are ideal for bone ingrowth, and a porosity of 60%–70% can significantly reduce the stiffness of a titanium implant, making it more similar to bone. The regenerative implant leverages these findings by incorporating a lattice that not only reduces stiffness (thereby helping prevent stress shielding). Nevertheless, it also provides a supportive environment for cells.

Furthermore, the use of hematopoietic stem cells is significant. These bone marrow-derived stem cells can differentiate into blood and immune cells, but the local microenvironment (niche) can also encourage mesenchymal differentiation. The marrow niche likely sends signals that could improve bone repair.

The implant essentially acts as an extension of the bone’s natural healing system, providing a scaffold through which marrow can flow (*via* internal channels connected to the intramedullary space) and settle to form new bone.

In practice, achieving proper regenerative integration requires more than a porous structure; it also requires a dynamic, responsive environment.

Potential improvements could involve bioactive coatings that attract stem cells or growth factors to boost osteogenesis. For instance, coating the pores with calcium phosphate (to mimic bone mineral) or loading them with bone morphogenetic proteins (BMPs) could enhance the regenerative response.

Some have proposed using gene therapy or cell-seeding techniques—such as harvesting a patient’s stem cells, expanding them, and then infusing them into the implant during surgery. However, this increases complexity (Gavette et al., 2024).

The current design emphasizes endogenous cell recruitment, a straightforward yet refined approach that guides the body’s natural healing process directly into the implant *via* its architecture. Preliminary animal studies or *in vitro* tests will be required to verify marrow infiltration into the implant and the formation of new bone within it.

Standard techniques, such as histological staining and micro-CT, enable quantitative visualization of bone ingrowth within porous implants *in vivo*, as demonstrated in large-animal models, including the sheep femoral defect model (Wauthle et al., 2015).

### Recruitment mechanisms

The recruitment mechanism functions through two distinct strategies to promote endogenous cell homing: architectural optimization and bioactive augmentation. Architectural components—such as pore size, porosity, and channel interconnectivity—can guide the migration of marrow-derived progenitor cells by enabling perfusion pathways, nutrient diffusion, and localized mechanical cues (Abbasi et al., 2020; Lu et al., 2023).

Bioactive augmentation, on the other hand, includes mineral or peptide coatings (e.g., CaP, RGD) and the controlled release of osteogenic or angiogenic factors such as BMPs, VEGF, and SDF-1α, which create chemotactic gradients that attract CXCR4+ cells and support early vascularization (Botterill and Khatkar, 2022; Joshi et al., 2023; Thevenot et al., 2010). Peri-operative strategies such as BMAC or MSC enrichment may further enhance early biological activity at the implant–bone interface (Hennessy et al., 2008; Hu and Olsen, 2016; Imam et al., 2017; James et al., 2016; Liu et al., 2020).

From a translational perspective, this architectural approach emphasizes simplicity to lower costs and regulatory hurdles, reduce mechanical failure risks, and improve surgical workflow efficiency (Karageorgiou and Kaplan, 2005; Li et al., 2016; Schulze et al., 2023; Taniguchi et al., 2016). While the osteogenic response might be slower than those of factor-loaded or cell-enhanced strategies, this approach avoids complex safety issues by relying on the reliable, long-term guidance of host biology.

In contrast, the use of BMPs or complex coatings as supplementary treatments often results in higher costs, operational difficulties, and potential adverse reactions, such as ectopic bone formation, which complicate their clinical use (James et al., 2016; Nikam et al., 2025). These issues mainly stem from the uncontrolled diffusion of BMP-2 beyond the target defect site, causing off-target osteoinduction and inflammatory signaling that are hard to regulate *in vivo* (Tannoury and An, 2014).

**FIGURE 2:**
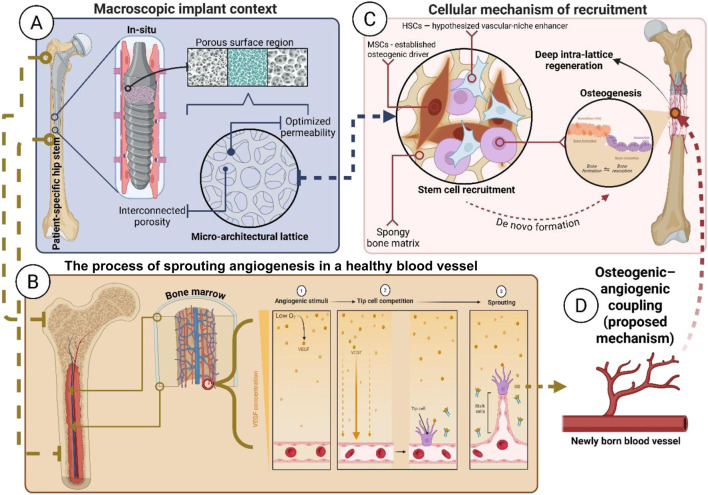
Multi-scale mechanism of architecture-driven endogenous regenerative integration. The schematic illustrates the proposed translational sequence: from macro-porous placement **(A)** to micro-lattice tuning **(B)** to stem cell recruitment **(C)** to osteogenic–angiogenic coupling **(D)**. Each panel description is as follows: **(A)** Macroscopic Context - View of the patient-specific hip stem, highlighting the proximal porous region targeted for biological fixation; **(B)** Micro-Architecture - Detail of the interconnected lattice structure (e.g., gyroid TPMS), optimized for permeability and marrow access; **(C)** Cellular Recruitment - MSCs (established osteogenic drivers) and HSCs (hypothesized vascular-niche enhancers) migrate from the host marrow niche to colonize the porous struts; and, **(D)** Proposed Coupling Mechanism - Recruited HSCs support neovascular formation, creating the vascular supply necessary to sustain MSC-driven osteogenesis and facilitate deep, vascularized biological integration.

A schematic illustration of this architecture-driven endogenous recruitment process—showing the essential movement of stem cells from the host marrow niche toward the implant’s porous struts, where osteogenic–angiogenic coupling begins—is depicted in Figure 2.

To validate this regenerative mechanism, a two-stage investigative framework is proposed: first, demonstrating architectural-element-based endogenous homing, followed by factorial studies using minimal adjuncts (such as a thin mineral layer or an ultra-low-dose localized factor) to optimize the biological response only when necessary. This staged approach allows architecture alone to be evaluated before testing whether small, controlled biochemical cues can amplify cell recruitment without introducing the safety risks associated with higher-dose bioactive systems (Bose et al., 2012).

Quantitative endpoints should include assessment of cell ingress and vessel fraction *via* µCT and histology—standard quantitative metrics for evaluating *in vivo* bone ingrowth and microvascularization in porous implants (Bouxsein et al., 2010). In addition, push-out strength and remodeling markers will be assessed, as these correlate strongly with long-term fixation stability and mechanoadaptive integration in load-bearing environments (Li et al., 2021; Taniguchi et al., 2016).

If successful, the regenerative method could produce improved long-term outcomes. Rather than a static metal implant that risks loosening over time, it would bond biochemically to the skeleton, forming new bone and potentially even partially transforming into living tissue.

Ultimately, the goal is for the boundary between the implant and bone to become indistinct over time, evolving into a functional bio-composite interface. This concept parallels the high-fidelity osseointegration observed in dental implantology, where fixtures function analogously to natural tooth roots (Adell et al., 1981; Duyck et al., 2001). Such internal colonization is supported by preclinical evidence showing that interconnected lattice networks permit vascular penetration, marrow formation, and bone remodeling under physiological loading (Murr et al., 2010; Wauthle et al., 2015). Accordingly, the proposed design extends this principle by promoting deep marrow infiltration and osteoconduction throughout the porous scaffold.

From a biomechanical viewpoint, minimizing failure mechanisms is essential. While deep-bone ingrowth improves secondary stability by reducing the risk of fretting wear and aseptic loosening caused by excessive micromotion (>150 µm) (Pilliar et al., 1986), high-porosity lattice designs inherently involve a trade-off in structural integrity. As porosity increases, permeability and biological ingrowth increase, whereas strut thickness and load-bearing capacity decrease, thereby increasing the risk of localized fatigue failure under cyclic physiological loading (Jones et al., 2023).

Therefore, the lattice architecture must be optimized to ensure a fatigue limit that exceeds *in vivo* stress levels while maintaining sufficient permeability and pore interconnectivity to promote vascular and marrow infiltration (Arabnejad et al., 2016; Wauthle et al., 2015).

Recent computational and experimental studies have shown that triply periodic minimal surface (TPMS) lattices and graded structures can achieve this balance by distributing mechanical stress more evenly and reducing stress concentrations within the scaffold, thereby enhancing both fatigue resistance and biological performance (Fan et al., 2024; Li et al., 2022).

Clinically, particularly in cases such as RDOA with considerable segmental bone loss, attaining this mechanical–biological equilibrium enables the regenerative implant not only to restore immediate load transfer but also to progressively reconstruct bone stock. This provides sustained advantages in potential revision surgeries, as the patient may ultimately have more viable bone than at the time of the initial arthroplasty. However, the clinical translation of regenerative medicine into orthopedic implants is still in its early stages. Significant challenges include ensuring uniform cell colonization, which relies on proper nutrient delivery and removal of metabolic waste within the lattice core (Karageorgiou and Kaplan, 2005). Additionally, infection prevention is essential because the large surface area of porous scaffolds makes them more susceptible to bacterial adhesion and biofilm formation (Arciola et al., 2018).

Finally, maintaining structural strength remains a challenge, necessitating careful topology optimization, as previously discussed, to prevent mechanical failure.

Notwithstanding these challenges, the proposed approach represents a significant paradigm shift.

A review of the literature reveals that while many hip implants feature porous surfaces to facilitate osseointegration, designs specifically engineered to promote endogenous stem cell “homing” remain lacking (Baryeh et al., 2023; Ziaie et al., 2025).

Therefore, this work aims to close the gap between tissue regeneration approaches and load-bearing joint replacements, emphasizing the collaboration between bioengineering and orthopedics. Through this state-of-the-art review, we propose a standardized methodology to define the emerging concept of ‘Regenerative Design’ in THA. This framework lays the theoretical foundation for integrating advanced manufacturing with endogenous biological repair, outlining a novel trajectory that warrants rigorous future experimental validation.

### Current studies and alternative research

Current treatments for complex RDOA cases include intra-articular biologics such as PRP, BMAC, and MSCs, along with cell-augmented scaffolds, grafts, and smart delivery matrices or gene-activated constructs that provide osteogenic and angiogenic signals to targeted areas (Almutairi and Alazzeh, 2024; Chandrashekar, 2024; Furuichi et al., 2024; Gibbs et al., 2016; Hu et al., 2020; Xiong et al., 2023).

These treatments demonstrate potential for pain relief and partial tissue healing. However, their effectiveness is highly variable due to the brief survival of injected cells, rapid dilution of cytokines, and the lack of continuous mechanical support in load-bearing settings (Lameire et al., 2025; Siddiq et al., 2024).

Furthermore, their high costs, complex delivery requirements, and limited long-term stability in the aggressive osteolytic environment typical of RDOA—where accelerated bone resorption surpasses the regenerative capacity of biologic therapies—raise serious concerns about durability and translational feasibility.

Alongside these biological approaches, notable progress has been made in structural and material innovation. For example, the clinical use of 3D-printed porous tantalum has proven effective in complex hip preservation surgeries, providing a strong alternative for customized structural support (Peng et al., 2024).

Furthermore, research on alternative polymer-based scaffolds continues to advance, with recent studies highlighting the potential of high-temperature 3D-printed PEEK implants to match the mechanical properties of native bone while avoiding stress shielding (Li et al., 2026).

Unlike traditional methods, the proposed methodology incorporates regenerative capacity directly into the implant structure. By promoting new bone formation and improving long-term stability, this strategy aims to reduce the need for revision surgery and enhance the mechanical strength of the bone-implant system (Ziaie et al., 2024).

Primary translational hurdles include validating implant performance under the high-resorption, high-inflammation conditions characteristic of RDOA, mitigating infection susceptibility linked to the increased surface area and microtopography of porous lattices, and optimizing the inherent trade-off between material strength and porosity (Alkentar et al., 2023; van Hengel et al., 2023).

These challenges require demonstrating that lattice geometries can withstand accelerated osteolysis, resist biofilm formation, maintain fatigue resistance under cyclic physiological loading, and still allow vascular and marrow infiltration. Therefore, future research should focus on disease-specific preclinical RDOA models that replicate rapid subchondral collapse and aggressive osteolytic activity, followed by carefully controlled early feasibility trials.

Key quantitative endpoints should include qCT/DEXA for regional bone density; vascular and bone histometrics; implant migration and micromotion analysis *via* RSA or 3D fluoroscopy; and early revision-free survival rates, all of which are essential for establishing clinical durability before assessing therapeutic efficacy.

While this review focuses on implant design and regenerative integration, we acknowledge that advances also influence clinical outcomes through surgical technique, intraoperative handling of biologically active materials, and postoperative rehabilitation protocols, which are outside our primary scope. These additional factors directly impact early mechanical stability, vascular recruitment, and the biological microenvironment around the implant—elements vital for successful regenerative fixation but inconsistently discussed in the existing literature.

Despite increasing interest in enhanced recovery pathways, navigation-assisted placement, and biologically informed loading protocols, current research remains fragmented and lacks unified, disease-specific guidelines. Future studies should integrate implant design with standardized surgical workflows and rehabilitation strategies to ensure a holistic, reproducible continuum of care that supports regenerative implants.

## Regulatory standards and mechanical testing requirements

Because regenerative, 3D-printed, patient-specific hip implants combine architecture-driven porosity, individualized geometry, and biologically oriented performance claims, their regulatory evaluation extends beyond traditional THA qualification. In addition to meeting baseline Class III mechanical and material standards (e.g., ISO 7206, ISO 5832), these systems require evidence that porous architectures remain reproducible, fatigue-safe, and interface-stable, and that the imaging-to-design-to-manufacturing pipeline is traceable and auditable.

Accordingly, regulatory-grade validation must include defect-aware segmentation reliability, mesh integrity, AM process control and repeatability, and architecture-specific mechanical testing, supported by standardized acceptance gates such as those summarized in Table 4. In this context, Table 3 consolidates the specific validation metrics needed to turn these concepts from a theoretical framework into clinical readiness. By outlining quantitative acceptance thresholds—covering segmentation accuracy (e.g., Dice ≥0.90), mesh integrity, and porous-transport adequacy—this table creates a standardized quality-assurance checklist.

**TABLE 4:** Regulatory and compliance matrix for patient-specific additively manufactured (AM) hip implants (ASTM F2924-21, 2021; ASTM F3001-23, 2023; ASTM F3302-18, 2018; ASTM F2924-14, 2014; ASTM F648-22, 2022; ASTM F75-23, 2023; European Parliament and Council, 2017; International Organization for Standardization, 2018; International Organization for Standardization, 2019; ISO/ASTM 52900, 2021; U.S. Food and Drug Administration, 2023a; U.S. Food and Drug Administration, 2023b; U.S. Food and Drug Administration, 2023c; U.S. Food and Drug Administration, 2024a; U.S. Food and Drug Administration, 2024b; U.S. Food and Drug Administration, 2025).

Lifecycle stage	Standard/regulation	Coverage	Evidence/outputs
Materials	ISO 5832-3	Ti-6Al-4V alloy chemistry and mechanical properties	Mill certs; tensile/elongation; Gas Analysis (O/N/H) chemistry
	ASTM F3001/ASTM F2924	AM Ti-6Al-4V ELI/PBF Ti-6Al-4V specs and properties	Build coupons (tension/fatigue), HIP record, HT certs
	ASTM F75/ISO 5832-4, ASTM F648	CoCr/UHMWPE specs (alternatives)	Supplier certs + mechanical tests
Quality and risk	ISO 13485	QMS for medical devices	QMS cert/alignment; DHF/DHF trace
	ISO 14971	Risk management across the lifecycle	Risk file (FMEA), mitigations, R/B
	EU MDR 2017/745/21 CFR 820	Regulatory framework (EU/US)	Tech file/510(k)/*De Novo*/PMA; UDI plan
Imaging and data	DICOM/ISO 12052	Imaging data standard and interoperability	Raw DICOM + acquisition parameters; de-ID logs
	HIPAA/GDPR	Privacy and data protection (US/EU)	De-ID SOP, consent/IRB docs
Biocompatibility	ISO 10993 (series)	Cytotox, sensitization, irritation, systemic/local effects; chem. characterization	ISO 10993 test reports (incl. 10993–18); residuals per 10993–7
Cleanliness and sterility	ISO 19227	Cleanliness of surgical implants	Cleanliness/particulate, residue reports
	ISO 11137/ISO 11135/ISO 17665/ISO 11607	Sterilization (radiation/EtO/steam) and packaging	Sterilization validation; dose audits; packaging validation
Design controls	IEC 62366-1 (if instruments)	Usability engineering	UE plan and summative report
	IEC 62304 (if planning software)	Medical device software lifecycle	Software Verification and Validation (SW VandV), SOUP list, risk
Modeling and simulation	ASTM F2996	FEA good practice for hip stems	FEA report: convergence <5%, contact sensitivity, fatigue margins
	ISO 7206-4/-6/-8	Hip stem endurance/fatigue testing	Physical fatigue to ∼10M cycles; worst-case constructs
	ISO 14242-1/-2	Hip joint wear simulation	Wear reports (mm^3^/MC), gravimetric data
Additive manufacturing	ISO/ASTM 52900 (2021)	AM terminology and basic principles	Process definitions in Device Master Record (DMR); SOP references
	ASTM F3122/ASTM F3302	AM metals property eval./process control	PQP/PPQ, calibration, parameter lock, SPC
	U.S. Food and Drug Administration (2017)	Technical considerations for AM devices	Process validation, build records, in-process and final inspection
Preclinical and clinical	ISO 14155	GCP for device clinical investigations	Clinical protocol, monitoring, consent, AE reporting
	US/EU pathways → IDE/CDE (US); CE marking under MDR	Early access and market authorization routes	Approvals (IDE/CDE), NB interactions; PMCF plan
Post-market	MDR PMS/PMCF/21 CFR 803 (US MDR reporting)	Surveillance, vigilance, CAPA, registry follow-up	PMS/PMCF reports; vigilance filings; CAPA logs

This matrix summarizes the complete regulatory and standards landscape governing the lifecycle of custom metal AM hip implants. It maps each stage—from material selection and manufacturing controls to biocompatibility testing, sterilization validation, mechanical performance, clinical investigation, and post-market surveillance—to the corresponding ISO, ASTM, FDA, and EU MDR requirements. The table outlines the evidence, validation outputs, and documentation necessary to demonstrate safety, mechanical reliability, and regulatory compliance for a Class III load-bearing implant (International Organization for Standardization, 2018; International Organization for Standardization (ISO), 2019; ISO/ASTM 52900, 2021; U.S. Food and Drug Administration, 2023a; U.S. Food and Drug Administration, 2024a, U.S. Food and Drug Administration, 2025).

This protocol ensures a consistent, audit-ready evaluation of patient-specific designs, confirming that next-generation architecture-driven regenerative THA solutions meet the strict safety and reproducibility standards required by regulatory agencies.

### AM-specific challenges: residues and process control

A unique challenge for regenerative lattice implants is confirming the removal of manufacturing residues from tortuous porous networks (e.g., per ISO 19227), where traditional cleaning validation may be inadequate. Because AM mechanical performance is highly process-dependent, regulatory attention underscores the importance of comprehensive process validation (IQ/OQ/PQ).

Since patient-specific geometries cannot be destructively tested individually, manufacturers must rely on nondestructive testing (NDT)—such as industrial micro-CT—along with validated witness-coupon correlations to verify internal lattice continuity and material integrity without risking sterile devices (Gavette et al., 2024; Food and Drug Administration, 2017).

### Preclinical and interface validation

Beyond standard stem fatigue qualification, the integrity of the porous–solid interface must be demonstrated through shear testing (ASTM F1044) to mitigate the risk of delamination and lattice–core failure. Considering the proposed regenerative mechanism, large-animal *in vivo* validation is also expected to confirm the biological feasibility and safety.

Importantly, these studies should go beyond baseline biocompatibility to offer quantitative evidence relevant to the specific hypothesis, including histological and micro-CT confirmation of vascular infiltration and marrow formation within the lattice.

### Regulatory pathway strategy

From a strategic perspective, prioritizing a cell-free, architecture-driven approach provides a clear regulatory advantage. By stimulating biological activity through geometric and mechanobiological design—without the need for exogenous cells or pharmacological agents—the implant avoids being classified as a combination product. This enables development within the traditional medical-device pathway, reducing complexity relative to drug-eluting or cell-seeded systems while still supporting endogenous regenerative functions.

Ultimately, aligning rigorous standards for mechanical, manufacturing, and biological validation is essential to integrating advanced computational methods into clinical practice. Employing a straightforward regulatory framework—focused on validated additive manufacturing, defect-detection quality assessments, and architecture-based verification of natural integration—enables regenerative, patient-specific implants to demonstrate better the safety and consistency required for Class III procedures.

This compliance-centered framework minimizes translational risk and provides a consistent method for delivering personalized, biologically integrative solutions to complex hip conditions such as RDOA.

## Ethical, economic, and societal considerations

The clinical translation of regenerative medicine within THA introduces ethical complexities that extend well beyond technical feasibility. As emerging strategies integrate patient-specific design, advanced additive manufacturing, and biologically informed architectures, they challenge existing norms regarding patient safety, equitable access, informed consent, and societal responsibility (Gavette et al., 2024).

The following section outlines the critical ethical, socioeconomic, and sustainability considerations associated with this rapidly evolving paradigm.

The first-in-human application of regenerative or biologically informed hip implants carries substantial uncertainty, making transparent and comprehensive informed consent an ethical necessity.

Patients must be clearly informed that these implants are investigational technologies, with long-term performance, biological behavior, and complication profiles that remain incompletely understood, especially concerning the dynamic interactions between porous regenerative structures and host bone or marrow environments. The consent process should explicitly distinguish these innovative approaches from conventional THA, explain their rationale, and limit their use to clinical situations in which existing treatments have poor outcomes, such as severe bone loss in rapidly progressive osteoarthritis.

An additional ethical duty is to avoid the “therapeutic misconception,” in which patients may mistakenly view participation in an early-stage study as receiving proven treatment. To prevent this, clinicians must stress uncertainty, potential risks, and the exploratory nature of early trials. Therefore, early clinical use should occur only within regulated research settings—such as formal clinical trials or national device registries—ensuring that the individual patient risk is justified by its contribution to general medical knowledge and is monitored through transparent oversight. Beyond safety, the economic burden of personalized regenerative implants is a significant barrier to equitable clinical adoption.

The end-to-end workflow of patient-specific 3D-printed prostheses—including high-resolution imaging, digital segmentation, individualized design, and additive manufacturing—naturally incurs higher costs than standardized, mass-produced implants. This discrepancy raises significant concerns regarding distributive justice, as such technologies risk becoming accessible only to well-resourced health systems and socioeconomically privileged patient groups.

Accordingly, the ethical integration of regenerative and patient-specific THA requires deliberate strategies to prevent widening health disparities. These include improving manufacturing efficiency, optimizing digital automation, and reducing design-to-implant time to lower overall unit costs.

Moreover, current reimbursement systems are often poorly aligned with novel personalized devices that do not fit existing procurement categories. As Tack et al. (2016) highlight, sustainable implementation will require reimbursement frameworks that account for long-term value—particularly the potential reduction in revision surgeries for patients with severe bone loss—rather than focusing solely on upfront procedural cost.

Regulatory authorities face a core ethical dilemma between non-maleficence—safeguarding patients from harm—and beneficence, which urges providing timely access to innovations that could treat conditions otherwise deemed impossible. For regenerative or biologically informed hip implants, extended preclinical testing helps reduce uncertainty. However, it might unintentionally disadvantage patients with rapidly progressing diseases, such as RDOA, especially when traditional treatments are limited.

This dilemma has driven interest in adaptive or conditional approval frameworks (Eichler et al., 2012), in which early access is granted once safety and probable clinical benefit are demonstrated, with continued approval contingent on rigorous, ongoing evidence collection.

Nevertheless, this rapid development also imposes an ethical obligation on developers and researchers.

Conditional access is justified only when combined with robust, transparent post-market oversight, including comprehensive clinical registries, systematic adverse event reporting, and the publication of negative results alongside positive ones. This commitment to open data management ensures that emerging failure modes are identified swiftly and that the collective experience of early users helps protect future patients.

Ultimately, such transparency promotes both ethical governance and the responsible development of regenerative arthroplasty technologies. The deep biological integration sought in regenerative hip implants introduces psychosocial considerations that differ markedly from those associated with conventional prostheses.

As regenerative architecture encourages the growth of living tissue around and within the implant, the precise boundary between the device and the body becomes less clear, raising complex questions about bodily integrity, identity, and the patient’s sense of self. Some people might feel uneasy about a device that partly functions as biologically active tissue. Conversely, others might view such integration as an extension of the body’s natural healing capacity.

Therefore, preoperative evaluation should extend beyond physical eligibility to include a psychosocial assessment, ensuring that candidates have appropriate expectations, emotional resilience, and a clear understanding of the uncertainties associated with emerging regenerative technologies. In contrast, successful integration can greatly enhance psychological wellbeing by restoring mobility, independence, and quality of life, especially for patients whose conditions greatly limit daily activities.

As Gavette et al. (2024) emphasize, ethical evaluation should stay strongly patient-centered, matching the use of advanced reconstructive technologies with individual values, lifestyle goals, and psychological readiness.

The extended lifespan of regenerative or biologically integrated hip implants requires a shared, long-term management strategy involving patients, clinicians, manufacturers, and regulatory agencies. Continuous monitoring is crucial for identifying late-onset issues, such as unusual regenerative reactions (e.g., heterotopic ossification), specific degradation processes, and particle-induced osteolysis.

Despite the increasing complexity of these devices, significant ambiguity persists regarding long-term liability and financial responsibility in the event of failure. Figuring out how to distribute accountability among clinicians, manufacturers, and payers remains a complex ethical and policy issue.

Accordingly, ethical innovation in this field requires transparent planning for post-implantation contingencies, including precise mechanisms to address premature failure, revision requirements, or unexpected biological responses. Suggested models—such as performance warranties, structured revision support, or integrated monitoring programs—have been proposed to ensure that the economic consequences of technology underperformance do not unfairly burden patients (Garrison et al., 2013).

Establishing such frameworks is critical for maintaining public trust and ensuring the responsible translation of regenerative arthroplasty technologies.

The digital infrastructure needed for personalized regenerative implants depends on extensive processing of sensitive Protected Health Information (PHI), including high-resolution imaging datasets, computational anatomical models, and AI-generated predictive analytics. Therefore, data stewardship and cybersecurity become crucial ethical priorities in this emerging clinical ecosystem.

The growing dependence on cloud-based design platforms and interconnected AI workflows increases vulnerabilities related to data sovereignty, unauthorized access, and the potential re-identification of supposedly anonymized datasets (Kaissis et al., 2020).

To reduce these risks, following international regulations like the Health Insurance Portability and Accountability Act (HIPAA) (U.S. Department of Health and Human Services, 1996) and the General Data Protection Regulation (GDPR) (European Parliament and Council, 2016) is crucial, supported by strict de-identification standards, end-to-end encryption for all data transmissions, and clearly established informed-consent procedures for using patient data in algorithm development or future model training (Price and Cohen, 2019).

Ensuring robust digital governance is therefore fundamental to maintaining patient trust and safeguarding the responsible adoption of personalized, data-intensive regenerative technologies.

Ultimately, the scientific and engineering complexities inherent in regenerative and patient-specific implant technologies require an equally strong commitment to ethical stewardship. Proactively engaging diverse stakeholders—including ethicists, patient advocates, clinicians, and regulators—is crucial for anticipating and addressing potential sociotechnical challenges before clinical implementation.

The framework outlined by Gavette et al. (2024) for developing advanced prosthetics provides a valuable template for incorporating stakeholder input and forward-thinking policy in this evolving field.

Although the convergence of regenerative medicine and permanent implantation represents a significant technological frontier, its translation must remain firmly grounded in patient-centered values. Principles of equity, transparency, and epistemic humility should guide decision-making to ensure that technological sophistication does not supplant the primacy of patient wellbeing.

Such an approach is central to achieving socially responsible innovation and sustaining public trust as regenerative orthopaedics continues to advance.

## Future perspectives and potential impact

**FIGURE 3:**
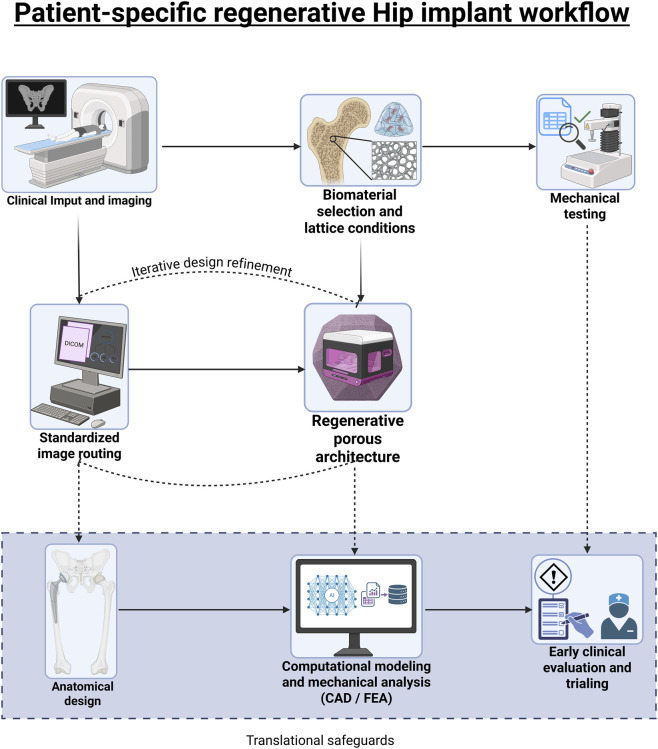
Proposed standardized workflow for patient-specific regenerative hip implant design. The framework is divided into three interconnected tracks: Primary design flow, which begins with clinical input and imaging, then moves through standardized image routing, biomaterial selection, and regenerative porous-architecture definition (based on the biological mechanisms in Figure 3), ending with mechanical and preclinical validation; Translational safeguards: which offers parallel checkpoints for anatomical design verification, high-fidelity computational modeling (CAD/FEA), and planning for early clinical evaluation; and, Iterative feedback: where curved arrows highlight the closed-loop nature of the workflow, showing how simulation and mechanical outcomes feed back into earlier stages to improve architecture and patient-specific fit. Overall, this demonstrates how regenerative feasibility and functional safety are co-optimized prior to clinical deployment.

To translate these regenerative design principles into clinical practice, a standardized, multidisciplinary workflow is crucial. The entire regenerative design lifecycle is summarized in Figure 3. Unlike linear workflow models, this diagram visually emphasizes the iterative, closed-loop nature of the proposed framework. It clearly highlights the integration of ‘translational safeguards'—key feedback loops where biological validation data (from Table 3) and computational insights re-inform the design parameters (from Table 2).

This visual representation shows how mechanical safety and biological performance are constantly balanced from the design to production continuum.

To translate these innovative regenerative principles into routine clinical reality, a standardized, multidisciplinary approach is essential. We propose a comprehensive methodological framework that integrates patient-specific anatomical design with rigorous translational safeguards—including iterative computational modeling and preclinical validation steps—to ensure both functional performance and patient safety throughout the development lifecycle.

The upcoming technological milestone involves transforming these traditionally passive devices into dynamic, data-driven platforms that can monitor their own biological and mechanical environments. Emerging research at the crossroads of additive manufacturing, micro-electromechanical systems (MEMS), and nanoscale sensing technologies indicates the potential for “smart” arthroplasty systems—implants that serve as both mechanical supports and continuous diagnostic interfaces.

Such systems could measure metrics such as micromotion, strain distribution, temperature changes, pH shifts, and early signs of inflammation, providing unprecedented *in vivo* insights into implant health (Ledet et al., 2018).

### Automation and AI

Beyond automated segmentation pipelines, the next significant step for patient-specific orthopedics is the development of Digital Twins: high-fidelity computational replicas of the patient–implant system that dynamically adapt to clinical data.

These virtual models combine imaging, material properties, gait dynamics, and physiological parameters to simulate implant performance under personalized loading conditions. Using high-throughput computing, digital twins can run large-scale Monte Carlo simulations and *in silico* clinical trials, testing thousands of virtual anatomies and activity profiles to predict failure modes, optimize lattice architecture, and improve fatigue resistance before fabrication (Viceconti et al., 2016).

This shift from reactive correction to proactive design optimization could notably accelerate innovation cycles. However, providing clinically reliable predictions requires comprehensive algorithm validation, transparent model calibration, and continuous monitoring for dataset bias—particularly with respect to underrepresented anatomies, activity levels, and pathological conditions.

Therefore, incorporating digital twin frameworks into regenerative implant development offers significant potential, provided that appropriate translational safeguards are in place.

### Beyond the hip

The methodological framework described in this review—combining patient-specific anatomical modeling with regenerative lattice structures—serves as a scalable platform technology with potential applications far beyond THA.

Anatomical regions with complex shapes or significant segmental bone loss, such as the spine, shoulder glenoid, pelvis, and extensive oncologic reconstructions, are especially suitable for this approach. Modern additive manufacturing platforms already enable the fabrication of custom trabecular or lattice structures for these applications; however, the primary challenge is whether natural cell-homing processes can be consistently replicated across different biomechanical settings.

A key research goal is to determine whether the pore gradients, permeability thresholds, and marrow-channel architectures optimized for the hip’s multi-axial, high-cycle loading environment are equally effective in regions dominated by compressive forces (e.g., vertebral bodies) or by tensile- and shear-dominant mechanics (e.g., shoulder and small joints). This question of mechanobiological universality is fundamental to scaling regenerative implant design throughout the musculoskeletal system.

Future breakthroughs will require comparative *in vivo* and *in silico* studies that map how regional stem-cell niches, marrow microenvironments, and joint-specific loading patterns influence endogenous recruitment, vascularization, and long-term remodeling. Addressing these gaps will determine whether the framework can develop into a unified design approach for reconstructive oncology, revision arthroplasty, and other complex surgical fields.

### Biological add-ons

While architectural optimization is the core strategy for regenerative implant design, emerging bioactive technologies represent a complementary research frontier that could improve regenerative outcomes in specific clinical cases (Kulkarni et al., 2024).

Future work in this area is best seen as a staged scientific roadmap rather than a strict set of design rules.

In the near term, research may focus on surface-level functionalization techniques, such as biomimetic mineral coatings or peptide motifs, such as RGD sequences. These simple modifications can facilitate early fixation without significantly altering the device’s regulatory profile, as supported by recent work showing improved osseointegration in RGD-functionalized porous titanium scaffolds (Rappe et al., 2022).

As investigations progress, more sophisticated biomaterial strategies—such as hydrogel-infused lattice networks or the spatiotemporally controlled release of osteogenic (BMP-2) and angiogenic (VEGF) cues—are promising ways to address impaired bone biology (Freeman et al., 2020). Additionally, cell-derived exosomes are emerging as a powerful acellular approach capable of modulating the immune microenvironment and accelerating mineralization (Zhai et al., 2020).

Long-term research directions might investigate the interface between additive manufacturing, gene-activated matrices (GAMs), and hybrid bioprinting techniques. Concepts such as mechanically responsive gene circuits or the *in situ* deposition of viable cell-laden bioinks into porous metal architectures demonstrate the potential for next-generation implants capable of coordinating controlled regenerative activity (Daly et al., 2016).

However, these high-complexity strategies also pose significant challenges related to biosafety, vector stability, manufacturing scalability, and regulatory oversight. Therefore, their translational viability depends on thorough preclinical evaluation and comparative studies that clearly balance benefits, risks, and costs.

Overall, these “biological add-on” approaches should not be regarded as immediate solutions but rather as future research directions that require systematic, evidence-based investigation. Their inclusion in this review is intended to promote critical scientific exploration rather than to endorse any specific clinical application.

### Smart monitoring

Building on predictive computational models, the physical integration of smart sensing technologies could be a future milestone in creating closed-loop feedback systems for arthroplasty. MEMS, passive RFID microsensors, and piezoelectric strain gauges have shown early promise for real-time monitoring of key physiological and mechanical parameters—including strain distribution, bone–implant micromotion, and local metabolic indicators such as pH or dissolved oxygen (pO_2_)—in real time (Ledet et al., 2018).

By detecting deviations in these signals, such systems could theoretically identify early signs of aseptic loosening, inflammatory dysregulation, or periprosthetic infection well before they appear on radiographs, thereby shifting postoperative management from reactive revision to proactive monitoring.

However, significant obstacles remain for clinical translation: sensor power longevity (e.g., *via* energy-harvesting techniques), signal accuracy through soft tissue, long-term biostability, and the secure transmission and management of sensitive biometric data. Overcoming these challenges will be crucial before smart monitoring systems can move from experimental prototypes to practical tools in regenerative implant care.

### Plausible future research focus

To move this paradigm from a theoretical framework to clinical application, future research should focus on three sequential, hypothesis-driven questions across three areas:

1. Foundational feasibility (pipeline validation).

Can the RFIS workflow be prospectively validated across multi-site RDOA CT collections to reliably generate defect-aware anatomical discrepancy maps and region-specific design constraints with acceptable inter-operator and inter-center reproducibility?

2. Biological plausibility (HSC pathway establishment).

In severe RDOA-like defects, does architecture-guided, marrow-accessible porosity enhance early vascularization and niche reconstitution while preserving established MSC-driven osteogenesis, consistent with a complementary HSC-supported mechanism?

3. Translational durability (fatigue performance).

What is the long-term fatigue performance of lattice–solid junctions under high-cycle, multi-axial loading representative of active RDOA patients, and which transition geometries most effectively reduce strut-level failure and interfacial delamination?

Together, these questions establish a logical validation sequence that advances from pipeline reproducibility to mechanobiological plausibility and ultimately to long-term translational safety.

## Conclusion

This review has synthesized current advances in patient-specific, additively manufactured hip implants and outlined a conceptual methodological framework that integrates regenerative design principles with individualized anatomical requirements.

Across the literature, drug-free, cell-free porous architectures have emerged as a promising strategy for leveraging endogenous marrow recruitment through hematopoietic stem cell–mediated osteogenic–angiogenic coupling. The convergence of modern imaging modalities, computational modeling tools (CAD/FEA), and Additive Manufacturing (AM) technologies provides a coherent foundation for exploring these biologically informed design pathways. Collectively, these approaches offer the potential to improve anatomical alignment, enhance early biological fixation, and possibly preserve or restore native bone stock in complex hip pathology.

However, despite encouraging mechanistic evidence, the long-term durability, reproducibility, and clinical permanence of such regenerative outcomes remain unverified and will require rigorous preclinical and clinical investigation. Bridging the gap between conceptual regenerative architectures and clinical practice will need a thorough, multi-layered approach.

Near-term priorities include enhancing manufacturing reproducibility and confirming mechanical and biological performance under worst-case loading conditions. Future efforts should involve disease-relevant large-animal studies to assess osseointegration dynamics, implant stability, and long-term lattice behavior within physiological environments. Only after meeting these foundational requirements can early human evaluation be ethically and scientifically justified under existing Class III regulatory frameworks.

Throughout this process, patient selection, transparent informed consent, and ongoing post-market surveillance remain vital safeguards to ensure that the potential benefits of these technologies are pursued responsibly.

Ultimately, the evidence summarized in this review emphasizes that the future of complex hip reconstruction depends not on a single breakthrough, but on the coordinated growth of multiple disciplines—specifically, the seamless integration of precision image routing, high-fidelity computational modeling, and biologically informed architectural design. While each component provides essential capabilities, none is sufficient on its own. The standardized methodological framework outlined here helps connect these fields, establishing a solid foundation for the next-generation of implant development.

In conclusion, the growing integration of these fields could transform how orthopaedic implants are designed, evaluated, and utilized, leading to more durable, biologically compatible, and patient-centered solutions in the future.

## Footnotes

1 Also known as rapidly progressive osteoarthritis.

2 Rare case of chondrolysis with unknown etiology.

3 Particle accumulation is attempted to be neutralized by the usage of inflammatory mediators.

4 Due to the frustrated immune response, the ongoing activation leads to inflammation and bone loss.

5 Working range for design comparisons.

6 Young modulus range depends strongly in anatomic site and apparent bone density.

7 Includes infections, neuropathic, osteonecrosis (AVN).

## Glossary

AM: Additive manufacturing
ASTM: American Society for Testing and Materials
BCE: Binary cross-entropy (loss)
Build coupons: Additive test specimens (tension/fatigue)
CAPA: Corrective and Preventive Action
CAD: Computer-aided design
CDE: (China) Center for Drug Evaluation (trial/device approval)
Clinical protocol/monitoring/consent/AE: Clinical study docs and Adverse-Event reporting
Cleanliness/particulate: Particulate/cleanliness residue reports
CT: Computed tomography
De-ID: De-identification
De-ID logs: De-identification audit logs
De-ID SOP: De-identification standard operating procedure
De Novo: FDA *De Novo* classification
DHF: Design History File
DHF trace: Requirements/tests traceability matrix
DICOM: Digital Imaging and Communications in Medicine
DMR: Device Master Record
Dose audits: Radiation sterilization dose verification
FEA: Finite element analysis
FEA report: Mesh/contact sensitivity and fatigue margins
FMEA: Failure Modes and Effects Analysis
FO: Femoral offset
FOV: Field of view
GO: Global offset
Gravimetric data: Mass-loss measurements
HD95: 95th-percentile Hausdorff distance
HIP: Hot isostatic pressing
HIP record: HIP process parameters
HT: Heat treatment
HT certs: Heat-treatment certifications
IEC: International Electrotechnical Commission
IDE: Investigational Device Exemption
IRB: Institutional Review Board
ISO: International Organization for Standardization
ISO 10993-18: Chemical characterization
ISO 10993-7: EtO residual limits
MAR: Metal artifact reduction
Mill certs: Mill/material certificates (heat/lot, composition, mechanicals)
MRI: Magnetic resonance imaging
NB: Notified Body
nnU-Net: Self-configuring neural network for segmentation
O/N/H: Oxygen/Nitrogen/Hydrogen content analysis
Packaging validation: Seal/transit/aging validation
Parameter lock: Controlled, locked manufacturing parameters
PMA: Premarket Approval
PMCF: Post-market clinical follow-up
PMS: Post-market surveillance
PQP: Process Qualification Plan
PPQ: Process Performance Qualification
Process defs (DMR): Manufacturing process definitions with SOP refs
IQ/OQ/PQ: Installation Qualification, Operational Qualification and Performance Qualification
QA: Quality assurance
QC: Quality control
QMS: Quality management system
QMS cert/alignment: ISO 13485/21 CFR 820 alignment
R/B: Risk–benefit analysis
Risk file: Risk management file (incl. FMEA and mitigations)
ROM: Range of motion
RDOA: Rapidly progressive osteoarthritis
SOP: Standard operating procedure
SOUP: Software of Unknown Provenance
SPC: Statistical Process Control
STEP: ISO 10303 neutral CAD format
STL: Stereolithography mesh
PLY: Polygon mesh format
SW V&V: Software verification and validation
Supplier certs + mechanical tests: Supplier CoC with measured mechanicals
Tech file: EU technical documentation (MDR)
Tensile/elongation: Tensile strength and elongation
510(k): FDA premarket notification
UDI: Unique Device Identification
UE: Usability Engineering (plan and summative report)
U-Net: Convolutional network for biomedical segmentation
Wear (mm3/MC): Wear rate in mm3 per million cycles
Δ: Change
µm: Micrometre
Ti-6Al-4V (ELI): Titanium-6% aluminum-4% vanadium (extra-low interstitial).
